# Structural basis of nucleosome recognition by the conserved Dsup and HMGN nucleosome-binding motif

**DOI:** 10.1101/gad.352720.125

**Published:** 2025-10-01

**Authors:** Jaime Alegrio-Louro, Grisel Cruz-Becerra, George A. Kassavetis, James T. Kadonaga, Andres E. Leschziner

**Affiliations:** 1 Department of Cellular and Molecular Medicine, University of California, San Diego, La Jolla, California 92093, USA;; 2 Department of Molecular Biology, University of California, San Diego, La Jolla, California 92093, USA

**Keywords:** nucleosome-binding motif, Dsup, high-mobility group N, nucleosome structure, 5S rDNA nucleosome

## Abstract

The tardigrade damage suppressor (Dsup) and vertebrate high-mobility group N (HMGN) proteins bind specifically to nucleosomes via a conserved motif whose structure has not been experimentally determined. Here we used cryo-EM to show that both proteins bind to the nucleosome acidic patch via analogous arginine anchors with one molecule bound to each face of the nucleosome. We additionally used the natural promoter-containing 5S rDNA sequence for structural analysis of the nucleosome. These structures of an ancient nucleosome-binding motif suggest that there is an untapped realm of proteins with a related mode of binding to chromatin.

## Abstract (precis)

In this study, Alegrio-Louro et al. used cryo-EM to structurally show that the invertebrate protein Dsup and the vertebrate protein HMGN interact with an active chromatin-bearing nucleosome through similar binding mechanisms. This work reinforces the biological significance of conserved, ancient nucleosome-binding motifs that underlie the evolution of protein–chromatin interactions.

The specific interaction of proteins with the nucleosome is central to the regulation of chromatin function and dynamics. However, our understanding of this aspect of chromatin regulation is limited by our knowledge of the basis of nucleosome recognition via conserved protein motifs. In this study, we investigated the interaction of the high-mobility group nucleosome-binding (HMGN) protein motif with the nucleosome. The canonical HMGN proteins are abundant factors that are present only in vertebrates, all of which have at least three *HMGN* genes (González-Romero et al. 2015; Nanduri et al. 2020). The HMGN proteins bind to two high-affinity sites on the nucleosome in a DNA sequence-independent manner via a conserved RRSARLSA motif (Mardian et al. 1980; Sandeen et al. 1980; Ueda et al. 2008). The interaction of this motif with the nucleosome has been examined by NMR and modeling (Kato et al. 2011), but its high-resolution molecular structure has not yet been experimentally determined.

Intriguingly, an HMGN-like motif is present in the tardigrade-unique damage suppressor (Dsup) protein, which is a nucleosome-specific binding factor that has been shown to protect fly, plant, yeast, and human cells from DNA damage (Hashimoto et al. 2016; Chavez et al. 2019; Kirke et al. 2020; Ricci et al. 2021; Aguilar et al. 2023; Zarubin et al. 2023). The HMGN-like motif in Dsup is essential for binding to nucleosomes and for the protection of DNA from damage by hydroxyl radicals (Chavez et al. 2019). At present, Dsup is the only nonvertebrate protein that has been found to bind to nucleosomes via an HMGN-like motif.

To understand the relationship between the nucleosome-binding motifs of Dsup and a canonical HMGN protein as well as to gain insights into their specific interactions with the nucleosome, we used cryo-electron microscopy (EM) to determine the structures of the Dsup–nucleosome complex and the HMGN2–nucleosome complex. In addition, to study nucleosome structure in the context of active chromatin, we analyzed nucleosomes that were reconstituted with a promoter-containing DNA sequence from a *Xenopus borealis* somatic *5S rDNA* gene (Rhodes 1985). These studies revealed that the 5S rDNA sequence is a useful alternative to the commonly used synthetic or α-satellite DNA sequences (Armeev et al. 2022).

## Results and Discussion

### Two Dsup molecules bind to the nucleosome

**Figure 1.**
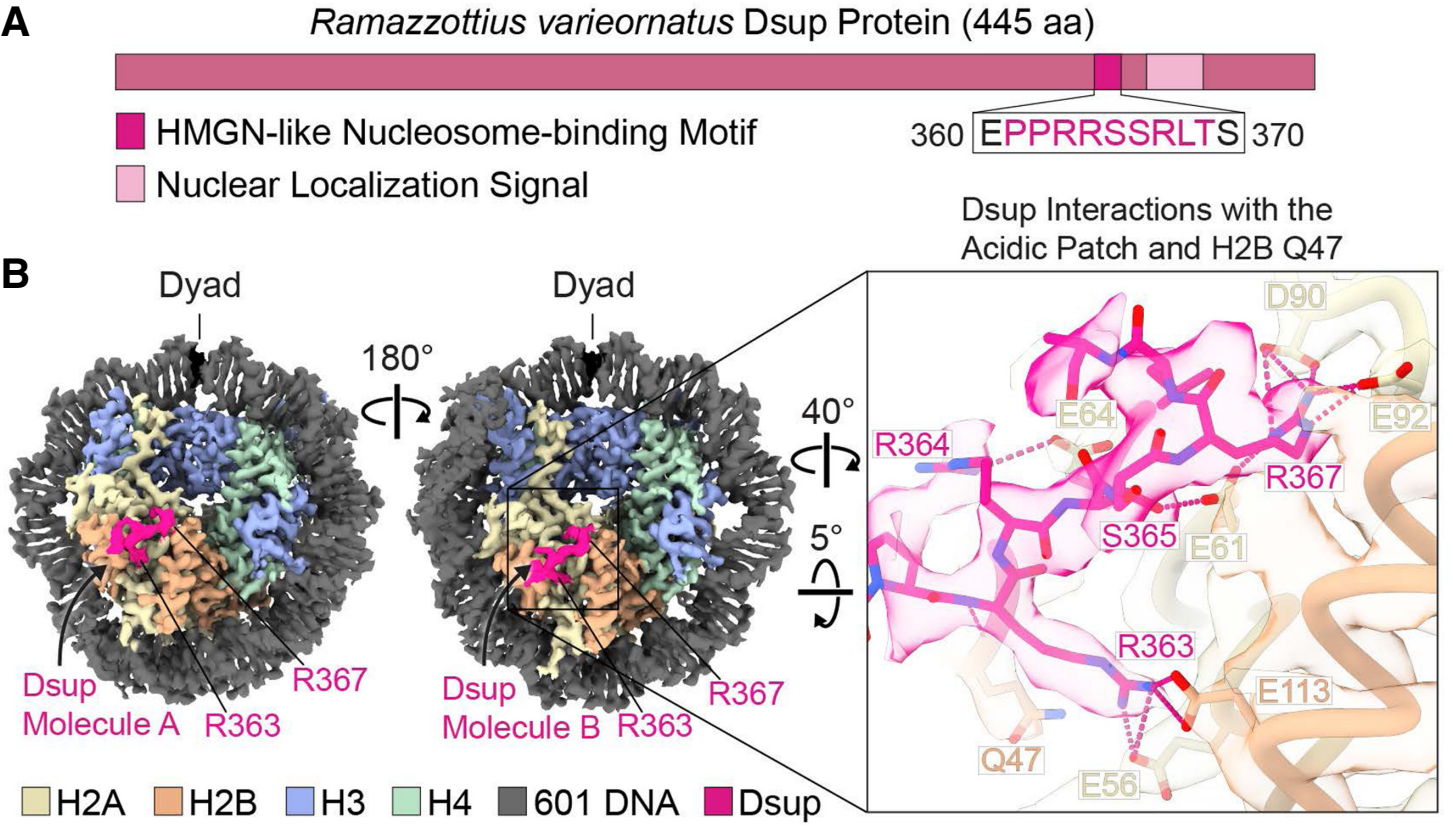
Two Dsup molecules bind to the nucleosome. (*A*) *R. varieornatus* Dsup protein. The HMGN-like nucleosome-binding motif of Dsup is highlighted (residues 360–370) (Chavez et al. 2019). The sequence in pink letters corresponds to the Dsup region modeled for each of the Dsup molecules in our structure. (*B*, *left*) Cryo-EM map of structure I (with one wrapped end and one unwrapped end) (Supplemental Fig. S2) for the Dsup-bound 147 bp 601 nucleosome cross-linked with glutaraldehyde. One Dsup molecule binds to each side of the disk. We refer to the two nucleosome-bound Dsup molecules as Dsup molecule A and Dsup molecule B. The close-up at the *right* shows the atomic model and the map in transparency, highlighting the interactions (dashed lines) of Dsup molecule B with the nucleosome acidic patch and with Q47 of the H2B α1–L1 elbow.

We initially sought to investigate the molecular interactions of Dsup with the nucleosome. To this end, we reconstituted the Dsup–nucleosome complex with recombinant *Ramazzottius varieornatus* Dsup (Fig. 1A) and the nucleosome core particle containing the 601 nucleosome-positioning sequence (Lowary and Widom 1998) and performed cryo-EM analysis. Data were collected from a single cryo-EM grid with a cross-linked sample. After 2D classification to discard poorly aligned nucleosomes, it was crucial to perform 3D refinement using an initial nucleosome reference with only one DNA end wrapped (and the other absent), thereby introducing asymmetry and mitigating the twofold averaging that otherwise results from the pseudo twofold symmetry of the nucleosome (Supplemental Fig. S1; Supplemental Movies S1, S2). To determine DNA sequence directionality in the cryo-EM maps, we modeled 25 bp of DNA centered in the nucleosome dyad in both possible orientations (related by a 180° rotation along the dyad axis) and assessed the confidence in the modeled atomic coordinates by comparing their B factors. This is an indication of how well the model accounts for the cryo-EM density in each of the two possible orientations (Supplemental Fig. S1; Supplemental Material). Two Dsup-bound nucleosome structures, which differ mostly in the dynamics of their DNA ends, were determined. In “structure I” (2.8 Å resolution), one DNA end is flexible, whereas in “structure II” (2.7 Å resolution), both ends are unwound (Supplemental Figs. S1, S2; Supplemental Table S1). Importantly, the two structures contain extranucleosomal densities that correspond to two Dsup molecules bound to either side of the disk (Fig. 1B; Supplemental Fig. S2). We refer to the Dsup molecules as “A” and “B” depending on whether they are bound to the face of the histone octamer proximal to the unwrapped or wrapped 601 DNA, respectively. The highest local resolution of the Dsup densities is 3.1 Å (Supplemental Fig. S1), which allowed us to model residues 361–369 of both Dsup molecules (Fig. 1; Supplemental Fig. S3). Notably, this segment contains an arginine-rich motif (RRSSR) that is part of the Dsup region that is similar to the core nucleosome-binding domain of the vertebrate HMGN proteins (Chavez et al. 2019). In addition, we obtained several 3D classes of Dsup–nucleosome complexes showing low-resolution Dsup densities in different locations, suggesting that Dsup can transiently adopt multiple conformations around the nucleosome (Supplemental Fig. S4). Hence, two Dsup molecules can bind to the nucleosome through their HMGN-like motif. Notably, mutations in this motif have been shown to disrupt the ability of Dsup to bind to nucleosomes and reduce DNA damage protection (Chavez et al. 2019).

### Dsup interacts with the nucleosome acidic patch

Most of the interactions of the HMGN-like motif of Dsup with the nucleosome are shared between structures I and II (Fig. 1; Supplemental Fig. S3). In both structures, the PPRRSSRLT segment of Dsup straddles the long α helix (α2) in H2A and has multiple anchor points on the surface of the nucleosome acidic patch and the H2B α1–L1 elbow (Fig. 1B; Supplemental Fig. S3). Our maps show clear densities for Dsup R363 and R367, providing details of their interactions with residues in H2A and H2B. The backbone of Dsup R363 and H2B Q47 interact via hydrogen bonding, whereas the side chain of Dsup R363 forms salt bridges with H2A E56 and H2B E113 (Fig. 1B; Supplemental Fig. S3). Furthermore, Dsup R367 interacts with H2A E61, D90, and E92 (with the exception of Dsup molecule A in structure II, in which E92 is pointing out) and thus functions as the arginine anchor that is found in most acidic patch-binding proteins (Fig. 1B; Supplemental Fig. S3; Barbera et al. 2006; McGinty and Tan 2021). Dsup R364 interacts with H2A E64 but was poorly resolved in one Dsup molecule, suggesting that it is more loosely attached than R363 and R367, which are buried within the acidic patch. In addition, Dsup S365 contributes to nucleosome binding through hydrogen bonding with either H2A E61 or H2A E64 (Supplemental Fig. S3). We also identified one class with Dsup density spanning the histone octamer (Supplemental Fig. S4A). In this class, the Dsup density extends from the N-terminal helix of H3 (H3 αN), the docking domain of H2A, and SHL +6.5 over the H2A–H2B dimer and then to the vicinity of helix 1 of H3 and H3 loop L1 of the second histone H3 molecule. Overall, these findings indicate that the binding of Dsup to chromatin involves interactions with the acidic patch as well as with other regions of the histone octamer.

### Two HMGN2 molecules bind to the acidic patch regions in the nucleosome

**Figure 2.**
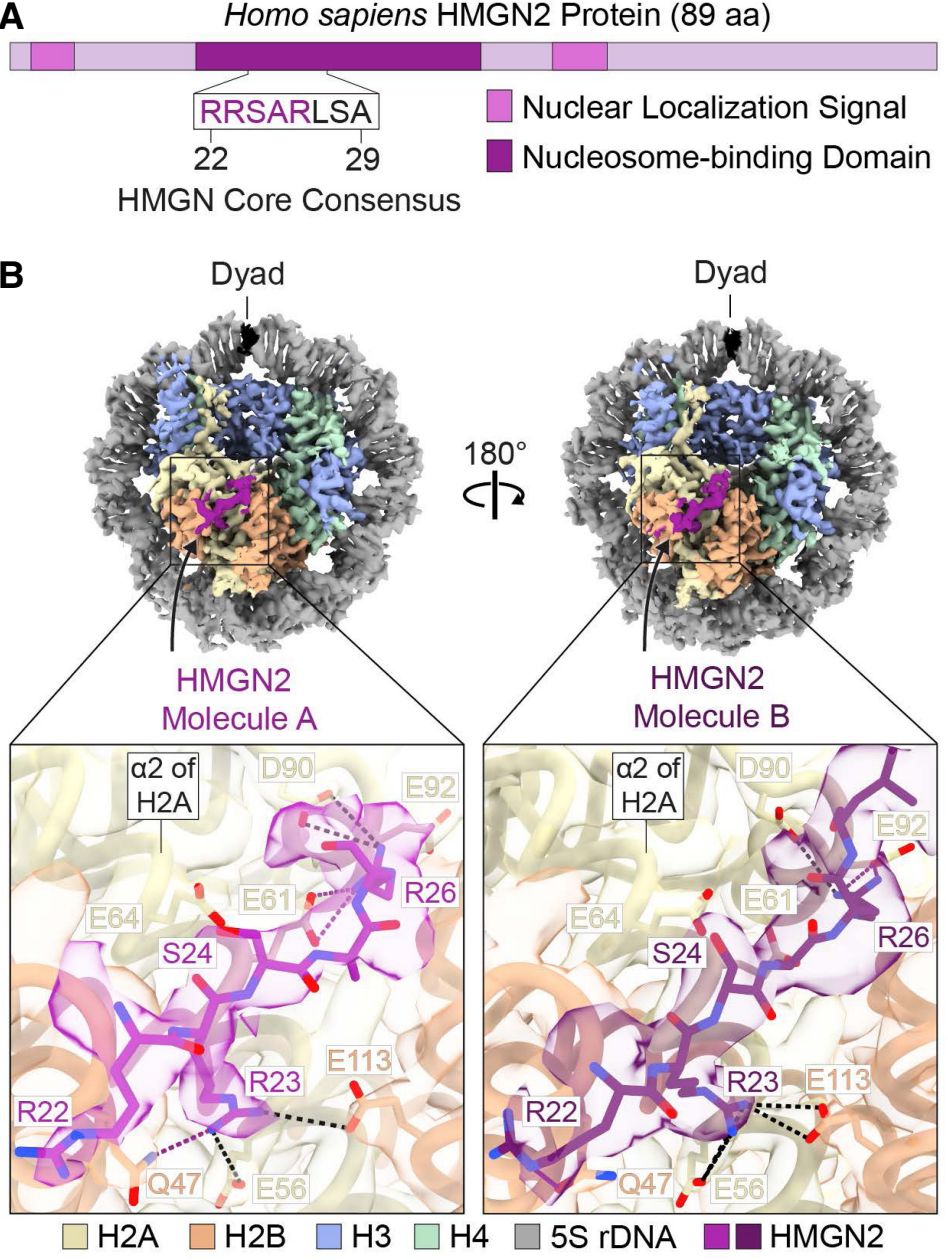
Two HMGN2 molecules bind to the nucleosome. (*A*) *Homo sapiens* HMGN2 protein. The sequence corresponds to the core consensus motif that anchors the HMGN proteins to the nucleosome (HMGN2 residues 22–29) (Ueda et al. 2008). The purple letters indicate the HMGN2 region modeled for each of the two HMGN2 molecules in our structure. (*B*) Cryo-EM map of the HMGN2-bound 167 bp 5S rDNA nucleosome. We refer to the two nucleosome-bound HMGN2 molecules as HMGN2 molecule A and HMGN2 molecule B. The binding of the two HMGN2 molecules to the nucleosome is not identical. The close-ups show the atomic model and the map in transparency, highlighting the shared (black dashed lines) and distinct (purple dashed lines) interactions of HMGN2 molecule A (light purple) and HMGN2 molecule B (dark purple) with the H2A–H2B heterodimers on opposite faces of the nucleosome.

We next expanded our cryo-EM studies to investigate the molecular interactions between a canonical HMGN protein and the nucleosome. To this end, we collected data from a cross-linked HMGN2–nucleosome sample containing human HMGN2 and a 5S rDNA nucleosome with 10 bp of linker DNA at each end. The 5S rDNA sequence contains the promoter region of a somatic *X. borealis 5S rDNA* gene. We obtained a 2.9 Å resolution map of the HMGN2-bound nucleosome (Supplemental Fig. S5; Supplemental Table S1). The map shows density for one full wrap of DNA around the histone octamer but lacks signal for the 10 bp linkers and most of the outer turns (Supplemental Figs. S5, S6), which suggests high DNA flexibility. Under our experimental conditions, the data analysis shows two HMGN2 molecules bound to the nucleosome, one on each face of the nucleosome at the acidic patch (Fig. 2B; Supplemental Fig. S6). The highest local resolution of the HMGN2 density on each side of the disk is 3.0 Å (Supplemental Fig. S5), including residues 22–26 of HMGN2, an arginine-rich sequence (RRSAR) that is conserved in all HMGN proteins (Fig. 2; Supplemental Fig. S6; Ueda et al. 2008; Chavez et al. 2019).

Both HMGN2 molecules use two major contact points in R23 and R26 at the ends of the acidic patch (Fig. 2; Supplemental Fig. S6). The binding of HMGN2 to the acidic patch is consistent with a proposed model for the interaction of HMGN2 with the nucleosome (Kato et al. 2011). Unexpectedly, however, our analysis revealed asymmetry between the two nucleosome-bound HMGN2 molecules. Specifically, although R23 in both HMGN2 molecules binds to the acidic patch through interactions with H2A E56 and H2B E113 (Fig. 2B; Supplemental Fig. S6), HMGN2 R26 interacts with H2A E61 and D90 in one HMGN2 molecule but with H2A E92 and D90 in the other HMGN2 molecule (Fig. 2B; Supplemental Fig. S6). Because HMGN proteins can bind to the nucleosome cooperatively (Postnikov et al. 1994), it is possible that the binding of one HMGN2 molecule affects the affinity or positioning of the second molecule at the acidic patch, leading to distinct HMGN2–histone H2A interactions. It is interesting to consider that this asymmetric binding could influence the nucleosome dynamics, potentially affecting the function of chromatin remodelers or transcriptional regulators. Hence, further studies are needed to explore the effects of this asymmetry in chromatin organization and gene expression.

Previous work proposed that the binding of HMGN proteins to nucleosomes decreases via serine phosphorylation within the RRSARLSA region (Prymakowska-Bosak et al. 2001). To understand the effect of this modification on HMGN2–nucleosome interactions, we modeled a phosphorylated version of HMGN2 S24 (pS24); this modeling predicted an electrostatic repulsion with the acidic patch that would prevent the interactions seen with the unmodified protein (Supplemental Fig. S7). Additionally, we solved the cryo-EM structure of HMGN5 bound to a nucleosome. We observed that nucleosome binding by HMGN5, which is the most divergent HMGN family member (González-Romero et al. 2015), appears to be similar to that by HMGN2 and primarily involves the RRSAR motif of HMGN5 (Supplemental Fig. S8). It is also important to note that AI-based predictions with AlphaFold 3 (Abramson et al. 2024) generate only low-confidence models of the HMGN2-bound nucleosome and do not identify the correct orientation of the region that binds to the acidic patch (Supplemental Fig. S9), as seen in our cryo-EM structure of HMGN2. Thus, HMGN–nucleosome complexes present a challenge for current AI-based structural prediction tools.

### The interactions of HMGN2 and Dsup with the acidic patch are related but not identical

The amino acid residues of the human histones comprising the nucleosome acidic patch are all conserved in *R. varieornatus* (Supplemental Fig. S10). The sequence and structural similarities between the nucleosome-binding motifs in Dsup and the HMGN proteins suggest a conserved mode of nucleosome recognition by these otherwise unrelated factors in *R. varieornatus* and humans, which have an estimated species divergence time of ∼700 million years (Kumar et al. 2022). In this regard, Dsup R367 and HMGN2 R26 are arginine anchors that interact with the H2A triad E61, D90, and E92 (Supplemental Fig. S6; McGinty and Tan 2021), and their positions within the nucleosome-binding domain align at the primary sequence level (Supplemental Fig. S6C). However, a distinction between the binding of HMGN2 and Dsup to the nucleosome is the use of a slightly different register for the insertion of the type 1 arginine (Dsup R363 and HMGN2 R23) into the acidic patch via interactions with H2A E56 and H2B E113 (McGinty and Tan 2021). These findings suggest some degree of functional refinement of the nucleosome-binding domains of these factors during evolution.

### The 5S rDNA nucleosome exhibits conformations with closed (wrapped) and open (unwrapped) DNA ends

**Figure 3.**
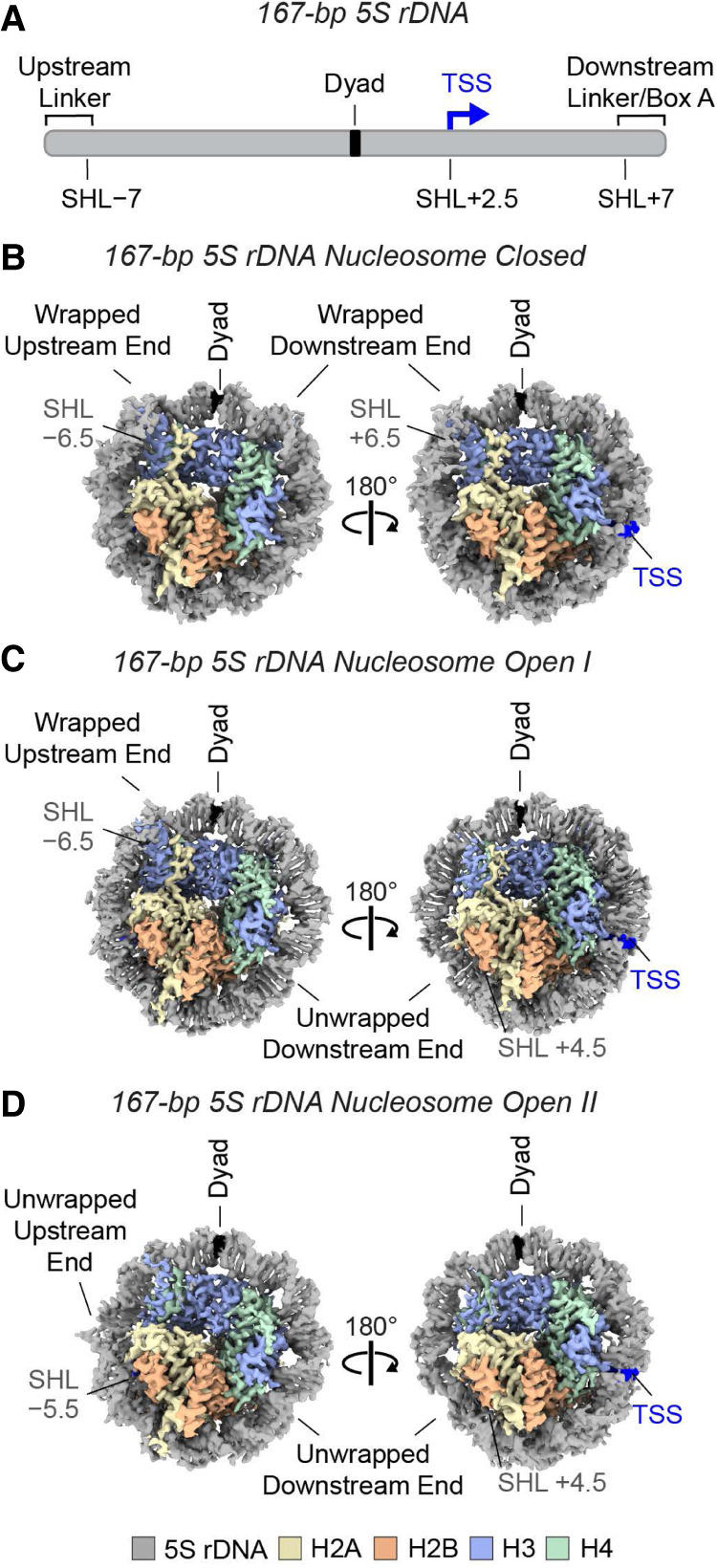
The 167 bp 5S rDNA nucleosome exhibits conformations with wrapped (closed) as well as unwrapped (open) DNA ends. (*A*) Schematic of the 167 bp 5S rDNA fragment containing 10 bp linker DNA on each side of the core nucleosome-positioning 5S rDNA sequence. The DNA ends are labeled as upstream or downstream relative to the transcription start site (TSS). The positions of superhelical location (SHL) −7 and SHL +7 are indicated. (*B*) Closed conformation of the 167 bp 5S rDNA nucleosome (both DNA ends wrapped). (*C*) Open I conformation of the 167 bp 5S rDNA nucleosome (one wrapped end and one unwrapped end). (*D*) Open II conformation of the 167 bp 5S rDNA nucleosome (both DNA ends unwrapped). *B*–*D* show cryo-EM maps.

To gain a better understanding of the conformation and dynamics of nucleosomes containing a natural DNA sequence that is involved in gene activity, we conducted a cryo-EM analysis of the 5S rDNA gene promoter nucleosome in the absence of HMGN proteins. We first obtained three structures from a non-cross-linked sample of the 167 bp 5S rDNA nucleosome, which contains a 10 bp linker DNA on each end (Fig. 3; Supplemental Figs. S11–S13; Supplemental Table S1). The three 167 bp 5S rDNA nucleosome structures, which we refer to as closed, open I, and open II, lack density for the linker DNAs and differ in the interactions involving the core nucleosomal DNA (Fig. 3; Supplemental Fig. S13). The closed 167 bp 5S rDNA nucleosome shows density for the entire 147 bp core DNA, which is wrapped around the histone octamer (Fig. 3A; Supplemental Fig. S13), and the open I and open II conformations show additional flexibility in one or both DNA ends, respectively (Fig. 3B,C; Supplemental Fig. S13). Thus, the 167 bp 5S rDNA nucleosome exhibits conformations with wrapped and unwrapped core DNA ends.

To assess the effect of the 10 bp linkers on the flexibility of the core nucleosomal DNA in these structures, we next analyzed a sample of the 147 bp 5S rDNA nucleosome (Supplemental Fig. S14). In the absence of linkers, the 147 bp nucleosome core particle adopts the closed and open I conformations seen with the 167 bp 5S rDNA nucleosomes (Supplemental Fig. S15). However, we did not observe a state analogous to open II, indicating that the flexibility of the core 5S rDNA increases in the presence of linker DNA. Notably, most nucleosomes in our 147 bp (∼60%) and 167 bp (∼95%) data sets adopted unwrapped conformations (Supplemental Figs. S11, S14; Supplemental Table S2), agreeing with the increased breathing dynamics of natural DNA sequences compared with the synthetic 601 DNA (Isaac et al. 2016).

### The DNA end that is downstream from the transcription start site (TSS) is preferentially unwrapped in the 5S rDNA nucleosome

Modeling of the 5S rDNA in the 147 and 167 bp nucleosome structures revealed that DNA unwrapping is more prominent on the end that is downstream from the TSS (Fig. 3; Supplemental Figs. S12–S15). First, the downstream end lacks density in both the open I and open II 5S rDNA nucleosome conformations (Fig. 3C,D; Supplemental Figs. S13, S15), whereas the upstream end exhibits high flexibility only in the open II state (Fig. 3D; Supplemental Fig. S13). Second, the lack of DNA density is greater at the downstream end (beyond superhelical location [SHL] +4.5 in open I and open II) relative to the upstream end (beyond SHL −6 in open II). Third, the preferential opening of the downstream DNA end is consistent with the higher number of particles observed in the open classes compared with the closed 5S rDNA nucleosome (Supplemental Figs. S11, S14). Finally, we did not detect nucleosomes that show flexibility exclusively at the upstream DNA end (Supplemental Figs. S11, S14). Increased flexibility in DNA that is downstream from the TSS may facilitate the transcription process, for instance, by exposing DNA binding sites for transcription factors (Sturges et al. 1995; White 1998).

Our nucleosome maps with high resolution in the octamer region revealed two arginines with different side chain conformations in the two H2A–H2B dimers of the 5S rDNA nucleosomes (Supplemental Figs. S16, S17). Specifically, R77 of H2A near the DNA end upstream of the TSS inserts into the minor groove at SHL −5.5, contributing to the stabilization of the DNA around the histone octamer in the closed, open I, and open II conformations. In contrast, in the two open states of the 5S rDNA nucleosomes, R77 of H2A near the downstream DNA end adopts a conformation that is not compatible with a fully wrapped DNA turn (Supplemental Figs. S16, S17). Interestingly, the loss of the interaction of H2A R77 with the minor grove has been correlated with the gain of DNA flexibility by molecular dynamics simulation and cryo-EM analysis of nucleosome core particles (Kniazeva et al. 2022; Ding et al. 2024). Furthermore, R86 of H2B facing the downstream outer gyre of the nucleosome establishes fewer contacts with the DNA than R86 in H2B on the opposite side (Supplemental Fig. S16). Thus, we observed asymmetries in H2A R77 and H2B R86 on the opposite faces of the 5S rDNA nucleosome that mirror the different flexibility of the DNA ends.

### Visualization of 5S rDNA nucleosome dynamics

Three-dimensional refinements and 3D classifications of the 5S rDNA nucleosome only describe the conformational dynamics with a few discrete states. To understand the more continuous nature of this flexibility, we performed principal component analysis (PCA) of the 167 bp nucleosome data set and generated 3D movies with the frames corresponding to the first two components (van Heel and Frank 1981; Punjani and Fleet 2021). PCA revealed that the main source of variability corresponds to the attachment/detachment of the downstream 5S rDNA end to/from the histone octamer. The transition between the closed conformation and the unwrapping of the downstream DNA end is indicated by the fading of the density between SHL +5 and SHL +7 and the increased mobility of H3αN and H2A residues 107–116 in some frames (Supplemental Movie S3). Consistent with this analysis, correlations between stabilization/flexibilization of these histone regions and DNA wrapping/unwrapping have been suggested for nucleosomes reconstituted in vitro and nucleosomes isolated from cells (Luger et al. 1997; Ferreira et al. 2007; Shukla et al. 2011; Arimura et al. 2021). The main component in the PCA also captures a bulge in the upstream DNA end and histone flexibility on the opposite side of the nucleosome (Supplemental Movie S3). The second component of variability in the data mostly involves the flexibility of the DNA upstream of the TSS, as indicated by a fully wrapped upstream DNA that progressively unwraps from SHL −7 to SHL −6, whereas the flexible downstream DNA, which lacks density between SHL +5 and SHL +7, partially rewraps around the histone octamer (Supplemental Movie S4). Altogether, the PCA is consistent with the preferential opening of the downstream DNA observed in the 5S rDNA nucleosome open I and open II structures.

### Cross-linking of the 5S rDNA nucleosomes results in DNA unwrapping

Glutaraldehyde (5 Å spacer arm) and formaldehyde (2–3 Å spacer arm) are often used to stabilize biomolecular interactions in cryo-EM samples containing nucleosomes (e.g., see Song et al. 2014; Lewis et al. 2021; Wang et al. 2021; Shioi et al. 2024). We set out to test their effects on the 5S rDNA nucleosome dynamics. Unexpectedly, cross-linking of the 147 and 167 bp nucleosomes with either glutaraldehyde or formaldehyde resulted only in cryo-EM structures of unwrapped nucleosomes (Supplemental Figs. S18–S21; Supplemental Table S2), which is in contrast to non-cross-linked samples that show both closed and open conformations. Furthermore, the cross-linked nucleosomes exhibit DNA that is flexible starting at SHLs ±5 (Supplemental Fig. S21), indicating greater flexibility in the upstream DNA end in cross-linked nucleosomes. We did not detect closed, open I, or open II states in either the 147 bp or 167 bp cross-linked nucleosomes (Supplemental Figs. S18–S21), which suggests that cross-linking affects the inherent dynamics of 5S rDNA nucleosomes. Moreover, the increased flexibility in the upstream DNA end in the cross-linked relative to the non-cross-linked nucleosomes correlates with a configuration of the side chain of H2A R77 that would collide with a fully wrapped DNA turn (Supplemental Fig. S22A). In addition, the two H2A R77 residues in the glutaraldehyde cross-linked 167 bp nucleosome adopt different conformations (Supplemental Fig. S22B) that are consistent with the highly flexible DNA ends. These findings support a relationship between the conformation of H2A R77 and the flexibility of the nearby nucleosomal outer gyre. Importantly, glutaraldehyde causes only a minor increase in the histone Cα root-mean-square deviation (RMSD) values (Supplemental Fig. S23), suggesting that octamer interactions not involving lysines, such as those of histones H2A and H2B with the HMGN motif, are largely unaffected by the cross-linking.

### Summary and perspectives

In this study, we used cryo-EM to visualize the interaction of the conserved Dsup and HMGN nucleosome-binding motif with the nucleosome. We first examined the tardigrade-specific Dsup protein, which we had previously found to be a nucleosome-binding protein with an HMGN-like motif (Chavez et al. 2019). This work revealed that the HMGN-like segment in Dsup binds to the nucleosome via the acidic patch (Fig. 1). We then performed cryo-EM with the human HMGN2 protein, which is a canonical HMGN protein that is found in all vertebrates (González-Romero et al. 2015), and saw that the HMGN nucleosome-binding domain interacts with the acidic patch in a manner similar to Dsup (Fig. 2). Notably, mutations within the acidic patch-interacting motif of HMGN2 and Dsup impair their binding to nucleosomes and disrupt their respective functions in chromatin regulation and chromatin protection (Ueda et al. 2008; Rattner et al. 2009; Chavez et al. 2019). The combined functional analysis, protein sequence, and structural data show that the HMGN nucleosome-binding domain is an ancient protein motif that is conserved from tardigrades to humans. It thus appears that the HMGN nucleosome-binding domain existed prior to the evolutionary split between protostomes and deuterostomes, and there may be an untapped realm of chromatin proteins with HMGN-like nucleosome-binding domains. During the course of this study, we identified a Dsup-like protein sequence containing an HMGN motif in the tardigrade *Paramacrobiotus richtersi* and found that a recombinant version of this protein binds to nucleosomes in electrophoretic mobility shift assays (Supplemental Fig. S24). These findings support the functional conservation of the HMGN motif across tardigrade genera. In the long term, systematic efforts involving functional, structural, and possibly synteny-based approaches to identify and characterize nucleosome-binding proteins across diverse lineages will be key to elucidating the evolutionary trajectory of the HMGN motif.

To understand the structure and dynamics of a nucleosome containing a natural DNA sequence that is involved in gene activation, we explored the use of the promoter region of the *X. borealis 5S rDNA* gene for our cryo-EM analyses of the HMGN2–nucleosome complex (Fig. 2) as well as of the nucleosome alone (Fig. 3). We observed conformational rearrangements that are consistent with nucleosome dynamics that facilitate transcription (Eltsov et al. 2018; Tan et al. 2023). The 601 sequence, which has an unusually high affinity for the histone octamer (Lowary and Widom 1998), can be readily reconstituted into nucleosomes but does not provide a good model for natural chromatin. Thus, for nucleosome structural studies, the 5S rDNA sequence is an appealing alternate to the 601 sequence. Our analysis, which showed that cross-linkers affect the conformations of the nucleosomal 5S rDNA, suggests that caution should be taken when drawing conclusions about DNA dynamics in samples involving cross-linked nucleosomes.

There remains much to be learned in the future. For instance, due to the generally high flexibility of Dsup and HMGN2 bound to the nucleosome, we have yet to identify any other key contact points between these proteins and the nucleosome, yet, at the same time, the flexibility of Dsup and HMGN2 on the nucleosome also likely explains why these abundant nucleosome-binding proteins are compatible with enzymes, such as DNA and RNA polymerases, that traverse nucleosomes. Understanding the effects of the Dsup and HMGN proteins on broader biological processes, such as development, remains an important area for future investigation. It would be interesting and informative to explore the properties of the HMGN-like nucleosome-binding domain beyond the canonical HMGN proteins. We also hope that the 5S rDNA nucleosome will be useful for future studies of chromatin structure, function, and dynamics.

## Materials and methods

### Cryo-EM sample preparation

The Dsup–601 nucleosome complex and the HMGN–5S rDNA nucleosome complexes were reconstituted essentially as described by Chavez et al. (2019), except that the nucleosomes contained recombinant human histones. Samples containing nucleosomes (in the absence of Dsup or HMGN proteins), Dsup-bound nucleosomes, or HMGN-bound nucleosomes were incubated with the cross-linking reagent (glutaraldehyde at 0.25% [v/v] final concentration for 5 min on ice or formaldehyde at 1% [v/v] final concentration for 10 min at 22°C; both reagents were in buffer HE: 25 mM HEPES-K^+^ at pH 7.6, 0.1 mM EDTA), and the reactions were stopped by the addition of 1 M Tris-HCl (pH 8.0) to 50 mM final concentration. SDS gel electrophoresis followed by Western blot analysis of the cross-linked samples showed that these conditions preserved the integrity of the complexes without detectable sample aggregation. In addition, non-cross-linked samples were prepared in parallel with buffer HE only in the reactions. The cross-linked and non-cross-linked samples were kept on ice prior to use in grid preparation for cryo-EM analysis. Additional methods are included in the Supplemental Material.

### Data and plasmid availability

Models and maps have been deposited in the Protein Data Bank and Electron Microscopy Data Bank under the following accession codes: 9D3K/EMD-46536, 9D3L/EMD-46537, 9D3M/EMD-46538, EMD-46539, 9D3O/EMD-46542, 9D3P/EMD-46543, 9D3Q/EMD-46544, 9D3N/EMD-46540, EMD-46541, 9D3R/EMD-46545, 9D3S/EMD-46546, and 9D3T/EMD-46547. Inquiries for the bacterial expression plasmids will be filled on request.

## Supplemental Material

Supplement 1

Supplement 2

Supplement 3

Supplement 4

Supplement 5

Supplement 6

Supplement 7
