# Supplementary material for: Structural basis of nucleosome recognition by the conserved Dsup and HMGN nucleosome-binding motif
Source: Genes Dev. 2025 Oct 1;39(19-20):1155–61. doi: 10.1101/gad.352720.125 (PMC12487698; doi:10.1101/gad.352720.125)
Supplement: Supplement 1 [file Supplemental_Material.pdf]

SUPPLEMENTAL MATERIAL

Structural Basis of Nucleosome Recognition by the Conserved Dsup and HMGN Nucleosome-binding Motif

Jaime Alegrio-Louro*, Grisel Cruz-Becerra*, George A. Kassavetis,
James T. Kadonaga, and Andres Leschziner

*These authors contributed equally to this work

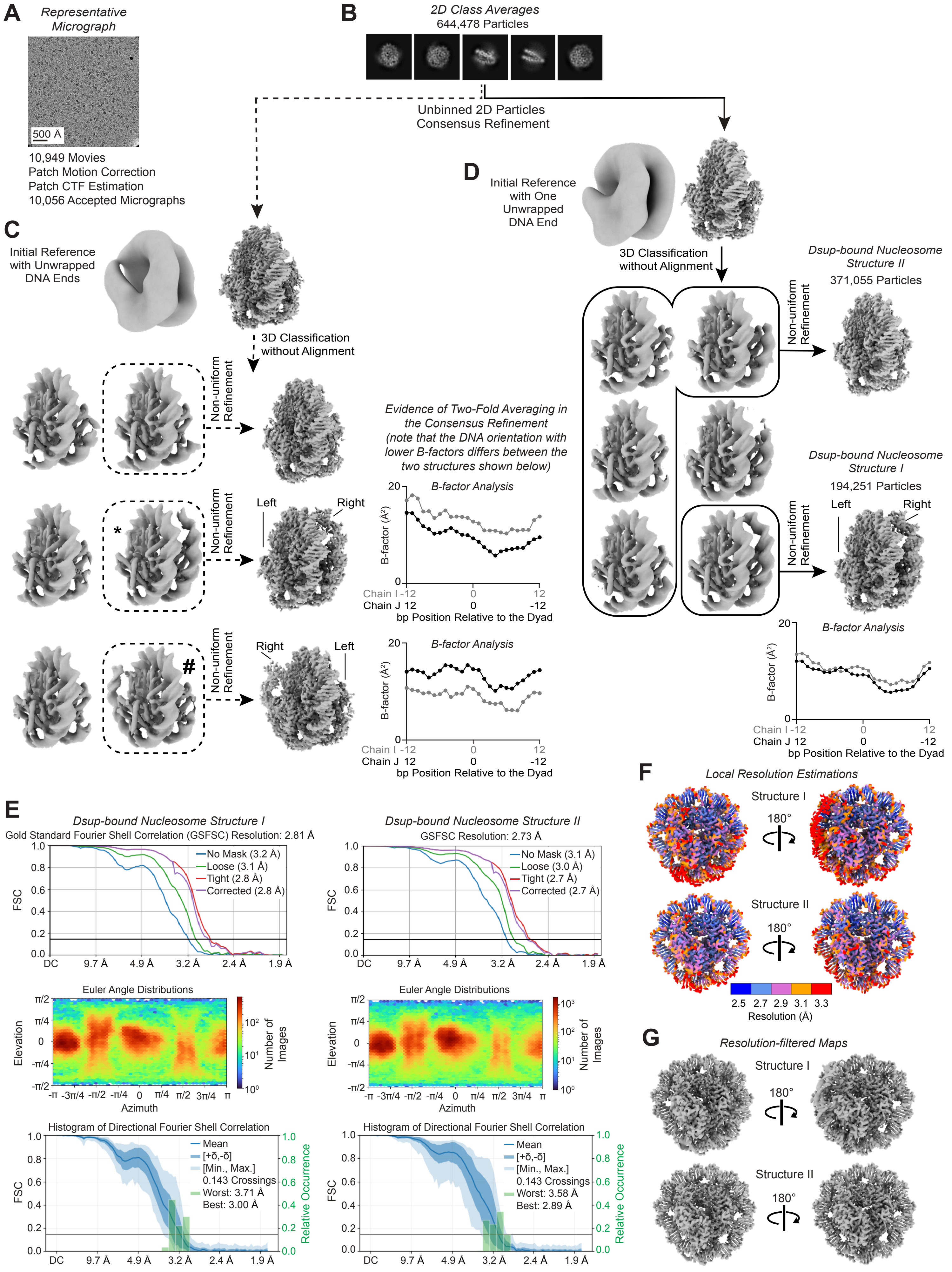

Alegrio-Louro et al. (Leschziner), Supplemental Fig. S1

Supplemental Figure S1. Image processing of the crosslinked Dsup-bound nucleosomes. Data collected from a single cryo-EM grid containing a crosslinked Dsup-nucleosome complex sample resulted in two structures, which we term structure I and structure II. (*A*) Representative micrograph, low-pass filtered to 10 Å. (*B*) Selected 2D class averages. (*C*) 3D refinement after 2D classifications using a low-pass filtered nucleosome with unwrapped DNA ends as the initial reference. Subsequent 3D classification without alignment shows classes with apparent differential flexibility in opposite DNA ends (indicated with * and #). Per-bp B-factor analysis of the two possible DNA orientations (lower B-factor orientation inverted between plots). (*D*) 3D refinement using a low-pass filtered nucleosome with one DNA end wrapped around the octamer as the initial reference. Subsequent 3D classification without alignment and per-bp B-factor analysis suggest preferential opening of the Dsup-bound nucleosome on the 'left' side, more precisely describing the dynamics in our dataset. DNA in structure I was modeled and deposited with the 'right' end wrapped. (*E*) Fourier shell correlations (FSCs), particle angle distributions, and histograms of directional FSC. (*F*) Local resolution estimation in Å plotted in five different colors onto the refined maps. (*G*) Maps filtered according to the local resolution estimations.

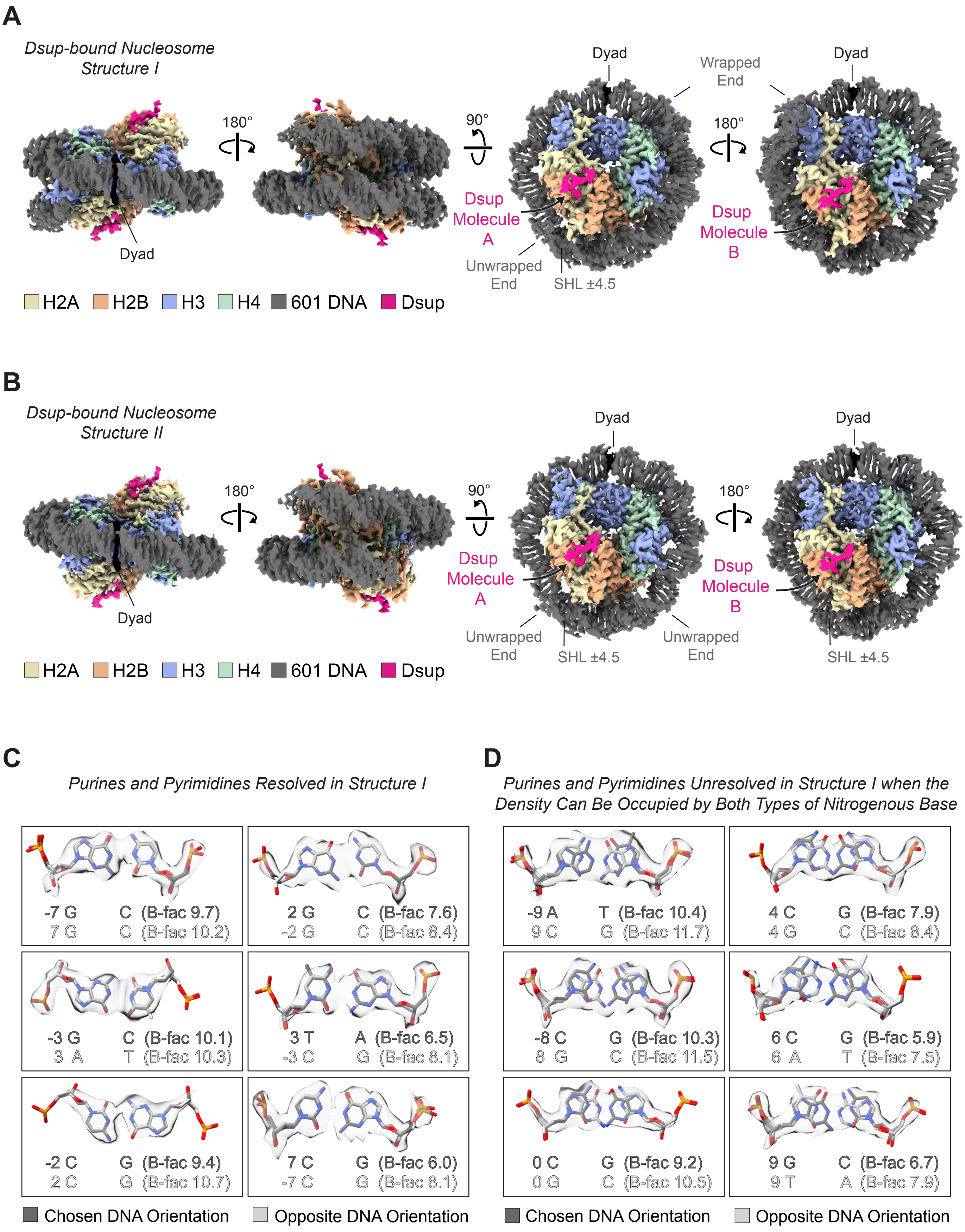

Alegrio-Louro et al. (Leschziner), Supplemental Fig. S2

Supplemental Figure S2. Cryo-EM maps of the Dsup-bound nucleosome structures. (*A*) Dsup-bound nucleosome structure I. (*B*) Dsup-bound nucleosome structure II. Each of the two structures shows density for two nucleosome-bound Dsup molecules (Dsup molecule A and Dsup molecule B). Structure I has one wrapped DNA end and one unwrapped DNA end. Structure II has both DNA ends unwrapped. SHL: superhelical location. (*C*) DNA bp densities that can be occupied by the same type of nucleotide (A/G or C/T) in both orientations (the one used to model the final DNA and its opposite), as well as their averaged bp factor. (*D*) DNA bp densities that can be occupied by different types of nucleotide (A/G in one orientation and C/T in its reverse), as well as their averaged bp factor.

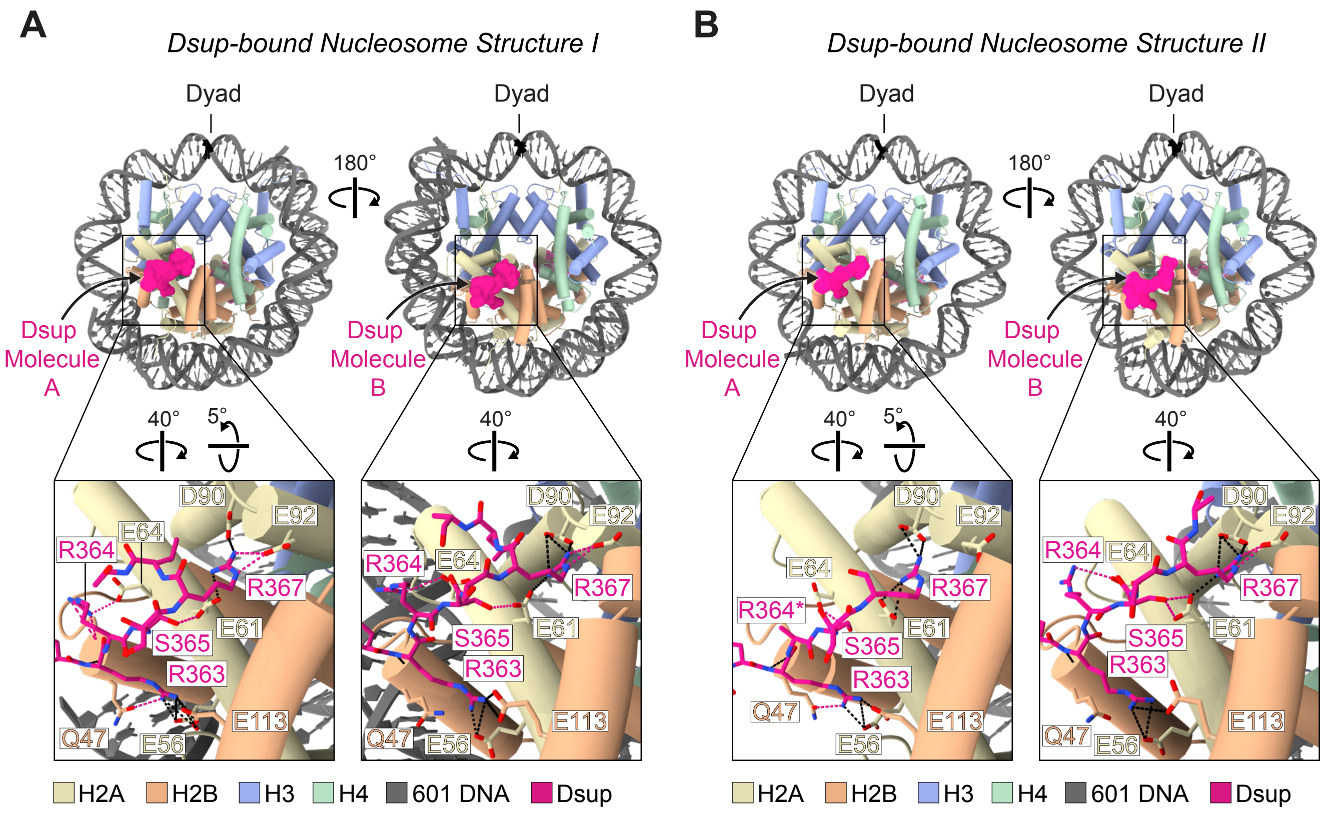

C

Summary of Dsup Interactions with the Acidic Patch and H2B Q47

	Dsup 363 R	Dsup 364 R	Dsup 365 S	Dsup 366 S	Dsup 367 R
**Structure I**					
Dsup Molecule A Interacts with:	H2A E56<br>H2B E113<br>H2B Q47#	H2A E64	H2A E61		H2A E61<br>H2A D90<br>H2A E92
Dsup Molecule B Interacts with:	H2A E56<br>H2B E113<br>H2B Q47	H2A E64	H2A E61		H2A E61<br>H2A D90<br>H2A E92
**Structure II**					
Dsup Molecule A Interacts with:	H2A E56<br>H2B E113<br>H2B Q47#		H2A E64		H2A E61<br>H2A D90
Dsup Molecule B Interacts with:	H2A E56<br>H2B E113<br>H2B Q47	H2A E64	H2A E61		H2A E61<br>H2A D90<br>H2A E92

Supplemental Figure S3. Interactions of Dsup with the nucleosome acidic patch and with Q47 of H2B. (*A*) Atomic model of the Dsup-bound nucleosome structure I. (*B*) Atomic model of the Dsup-bound nucleosome structure II. The black dashed lines in the inset images represent the interactions that are shared by Dsup molecule A and Dsup molecule B in structure I and structure II. The other Dsup interactions are depicted by pink dashed lines. The asterisk at R364 of one Dsup molecule indicates that the side chain is not modeled due to low resolution. (*C*) Summary of the interactions of Dsup molecule A and Dsup molecule B (residues 363-367) with the nucleosome acidic patch and with Q47 of the H2B α1-L1 elbow. The # symbol highlights the double hydrogen bonding between H2B Q47 and Dsup R363.

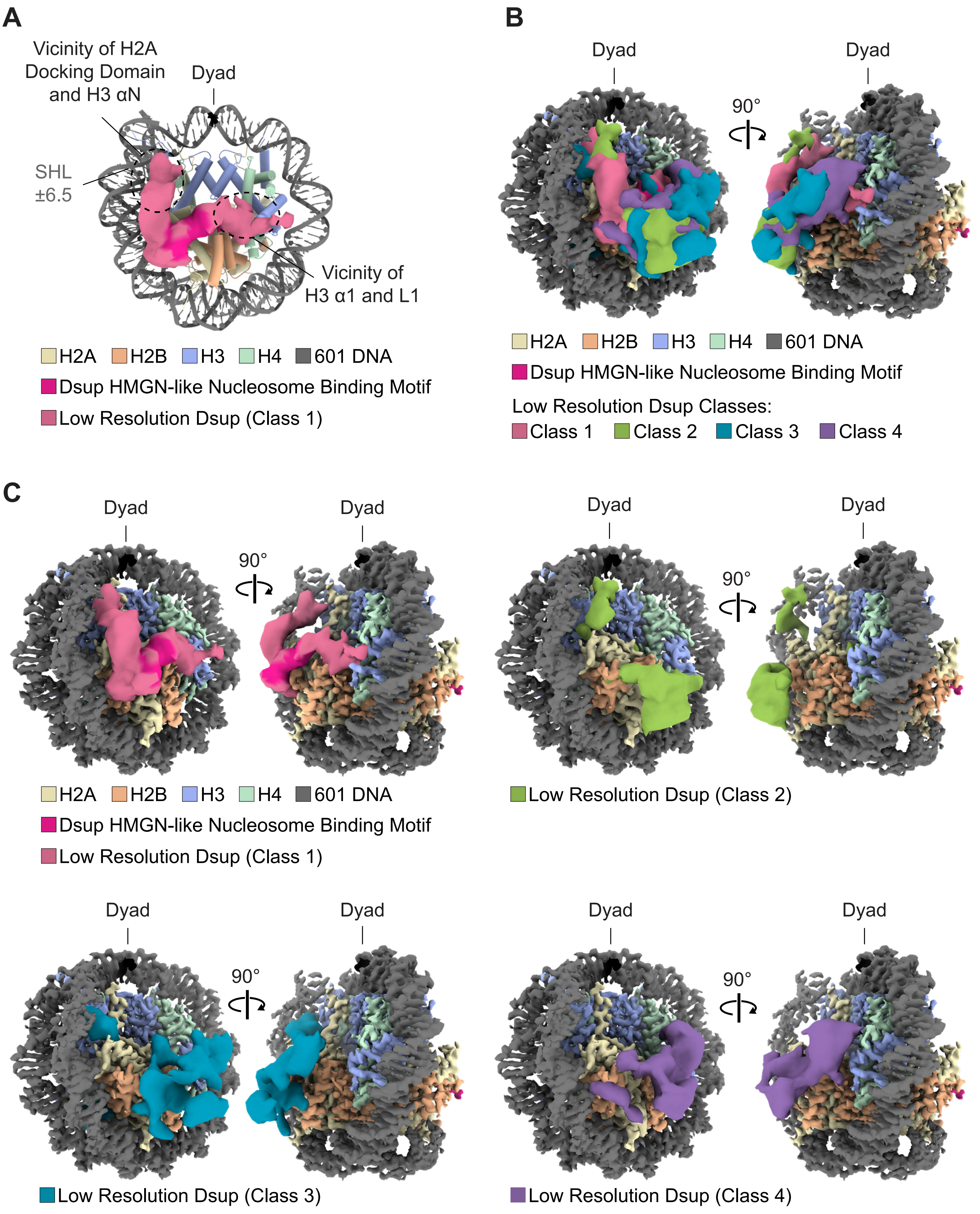

Alegrio-Louro et al. (Leschziner), Supplemental Fig. S4

Supplemental Figure S4. Dsup adopts different conformations beyond the nucleosome-binding core region. (*A*) Low-resolution Dsup density (class 1) obtained after nucleosome signal subtraction and focused classifications without alignment. SHL: superhelical location. (*B*) Overlay of four low-resolution electron microscopy Dsup classes. (*C*) Density maps of each of the four low-resolution Dsup classes.

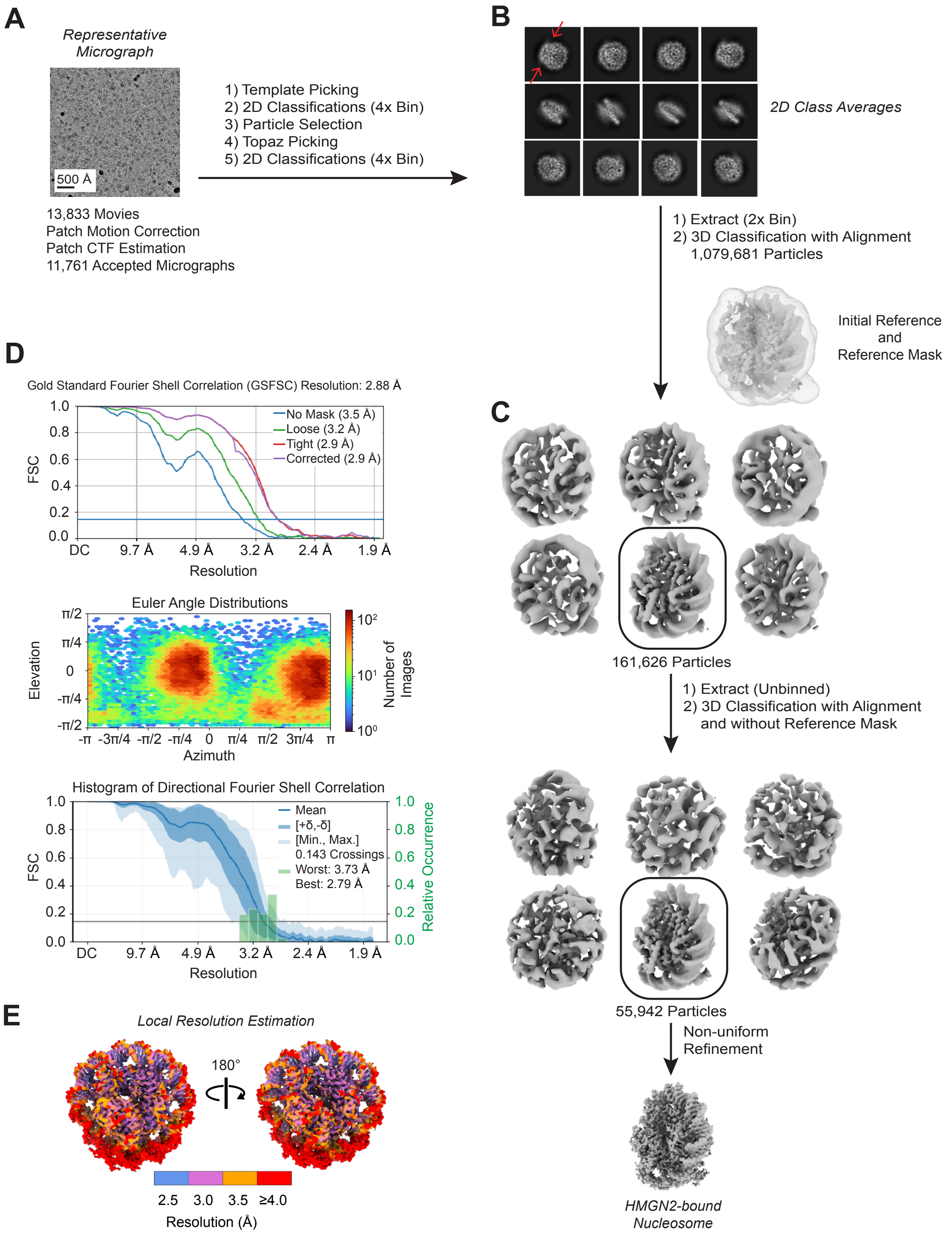

Supplemental Figure S5. Image processing of the crosslinked HMGN2-bound nucleosomes. (*A*) Representative micrograph, low-pass filtered to 10 Å. (*B*) Selected 2D class averages. The two red arrows indicate the flexibility (absence of density) of both DNA ends. (*C*) Image processing scheme. (*D*) Fourier shell correlation (FSC), particle angle distribution, and histogram of directional FSC. (*E*) Local resolution estimation in Å plotted in four different colors onto the refined map.

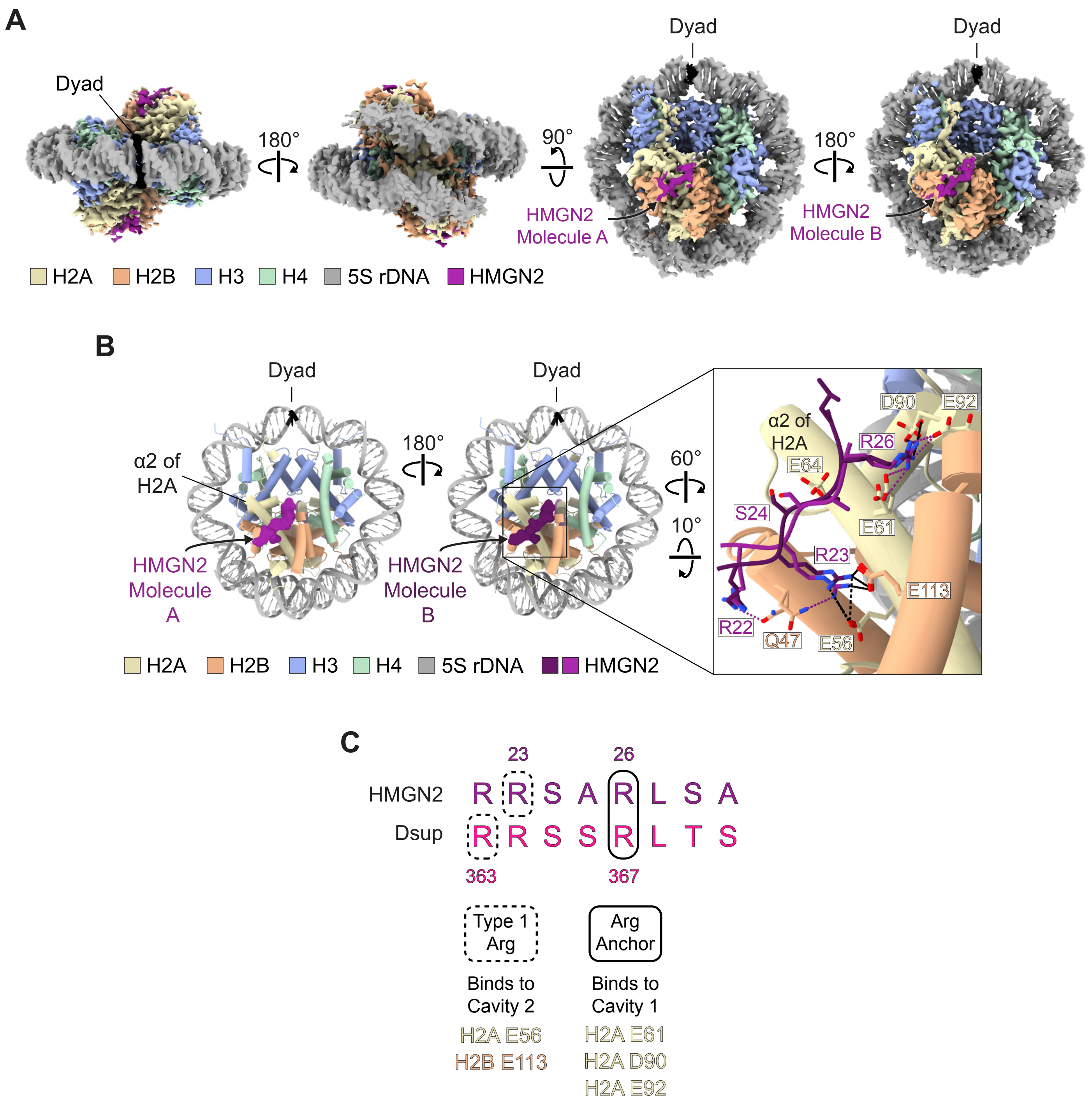

Supplemental Figure S6. Interactions of HMGN2 with the nucleosome acidic patch and with Q47 of H2B. (*A*) Cryo-EM map of the HMGN2-bound nucleosome structure. (*B*) Model of the HMGN2-bound 167-bp 5S rDNA nucleosome. The close-up highlights the interactions of HMGN2 molecule A and HMGN2 molecule B with the acidic patch and with H2B Q47. The black and purple dashed lines depict interactions that are shared and distinct, respectively, on each of the two faces of the nucleosome. (*C*) The interactions of HMGN2 and Dsup with the acidic patch are related but not identical. The HMGN2 and Dsup arginine anchor and Type 1 arginine show conserved and mismatched registers. Both the acidic patch and Q47 in the H2B α 1-L1 elbow are hotspots for tethering nucleosome-binding factors via interactions with arginine residues (McGinty and Tan 2021).

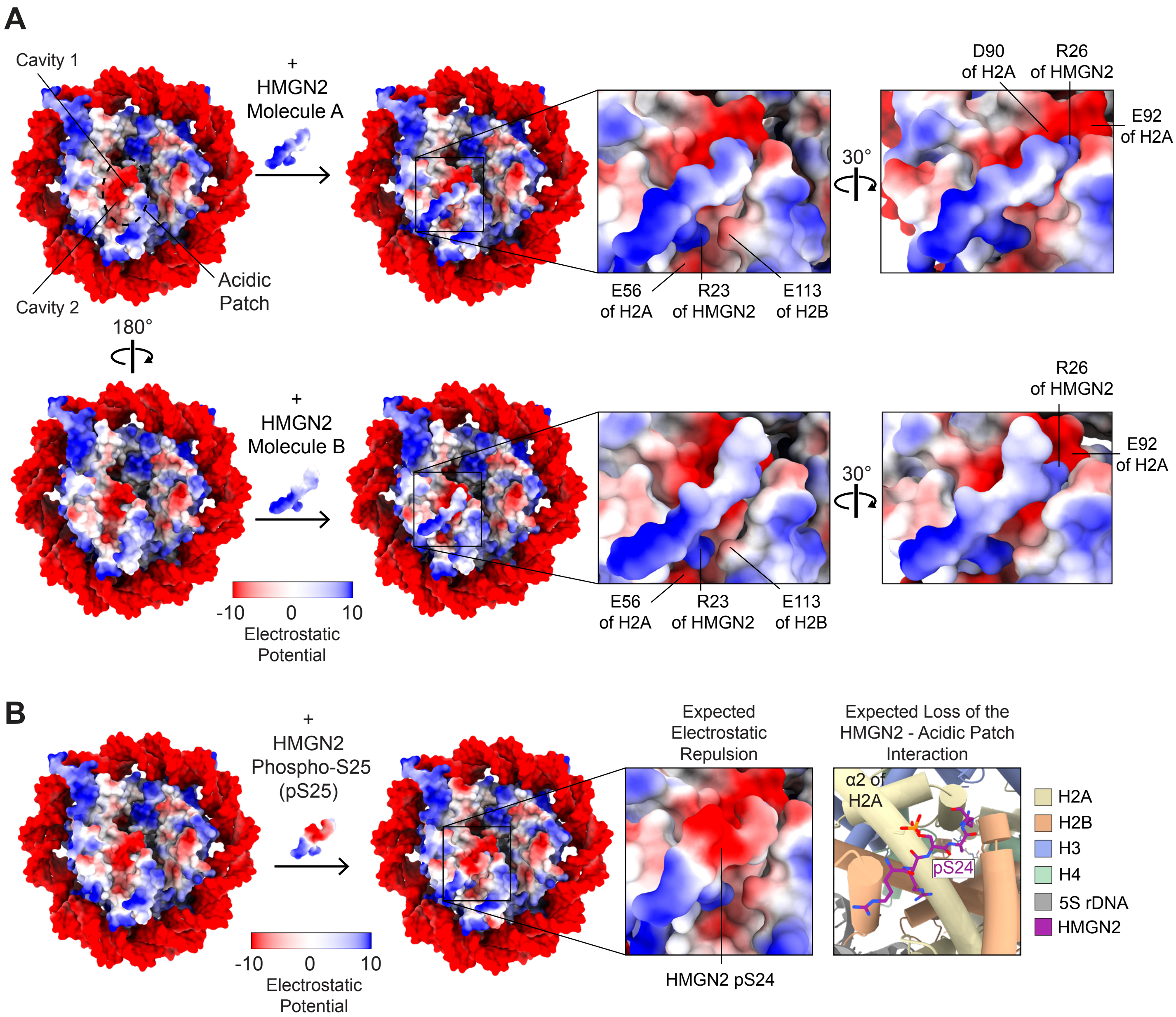

Supplemental Figure S7. Charge distribution maps of the HMGN2-bound nucleosome. (*A*) HMGN2-bound 5S rDNA nucleosome colored by Coulombic electrostatic potential. In the farthest left surface images, the densities for HMGN2 molecule A and HMGN2 molecule B are removed from the maps for clarity. (*B*) The RRSAR segment of HMGN2 was modeled with phosphorylated S24 (pS24) to show the anticipated electrostatic repulsion with the negatively charged acidic patch (compare panels *A* and *B*).

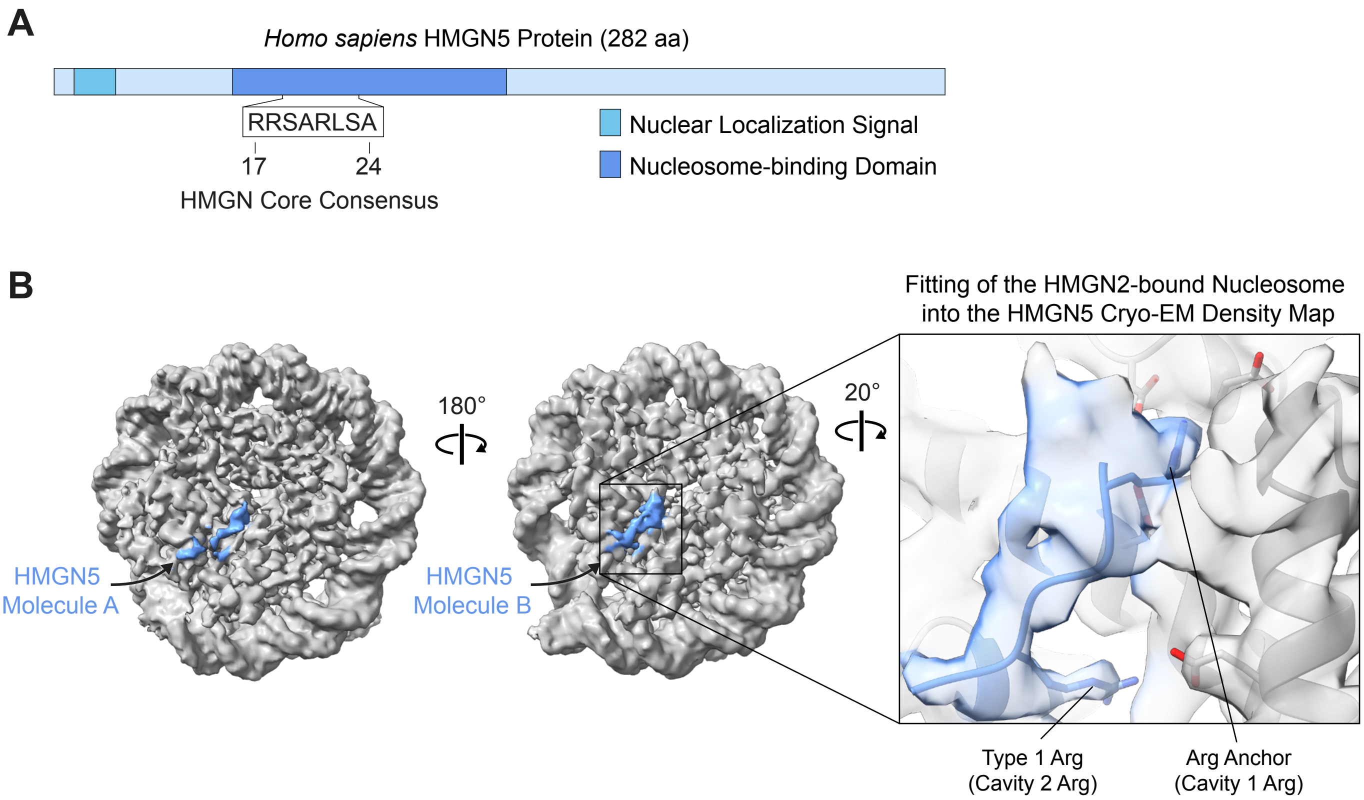

Supplemental Figure S8. Binding to the nucleosome by HMGN5 appears to be similar to that by HMGN2. (*A*) *H. sapiens* HMGN5 protein. The sequence corresponds to the core consensus motif that anchors the HMGN proteins to the nucleosome (HMGN5 residues 17-24). (*B*) Cryo-EM map of the HMGN5-bound 167-bp 5S rDNA nucleosome crosslinked with glutaraldehyde. The close-up view on the right shows the model of the nucleosome bound to HMGN2 fitted into the HMGN5-nucleosome map.

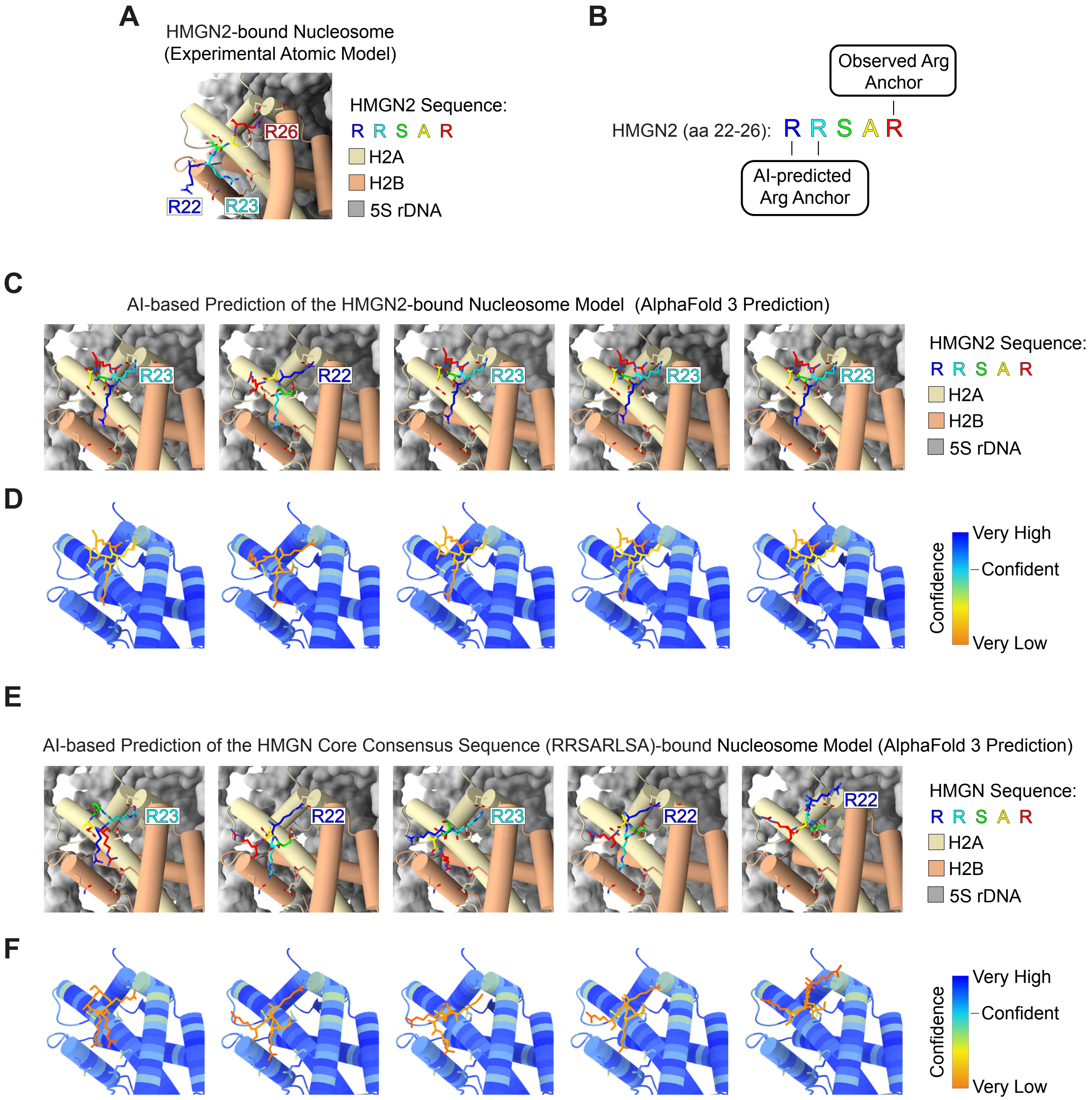
A
HMGN2-bound Nucleosome
(Experimental Atomic Model)
R26
R22
R23
HMGN2 Sequence:
R R S A R
H2A
H2B
5S rDNA
B
Observed Arg Anchor
HMGN2 (aa 22-26): R R S A R
AI-predicted Arg Anchor
C
AI-based Prediction of the HMGN2-bound Nucleosome Model (AlphaFold 3 Prediction)
R23
R22
R23
R23
R23
HMGN2 Sequence:
R R S A R
H2A
H2B
5S rDNA
D
Very High
Confident
Very Low
Confidence
E
AI-based Prediction of the HMGN Core Consensus Sequence (RRSARLSA)-bound Nucleosome Model (AlphaFold 3 Prediction)
R23
R22
R23
R22
R22
HMGN Sequence:
R R S A R
H2A
H2B
5S rDNA
F
Very High
Confident
Very Low
Confidence

Supplemental Figure S9. AI-based structural predictions do not correctly identify the experimentally observed arginine (Arg) anchor in HMGN2. (*A*) Experimentally determined structural model of HMGN2 bound to the nucleosome acidic patch. (*B*) Cryo-EM-observed and AI-predicted Arg anchor in HMGN2. (*C,D*) AI predictions of nucleosome-bound full-length HMGN2 with AlphaFold 3 (Abramson et al. 2024). (*E,F*) AI predictions of the nucleosome-bound HMGN core consensus sequence (RRSARLSA) with AlphaFold 3. In panels *A*, *C*, and *E*, only the RRSAR sequence [rainbow-colored from N- to C-terminus (blue to red)] is shown in the models. Panels *D* and *F* display the per-residue accuracy of the models shown in *C* and *E*, indicated by the predicted Local Distance Difference Test (pLDDT): very low (< 50, orange), low (50-70, yellow), confident (70-90, cyan) and very high (> 90, blue). The predictions are mostly very high confidence for the histones and very low confidence for the HMGN motif.

A

```
Hs H2A     0   MSGRGKQGGKARAKAKSRSSRAGLQFPVGRVHRLLRKGNYAERVGAGAPV    49
               |:|||| |||.:.|:|||||||||||||||:||:||||||||||||||||
Rv H2A     0   MTGRGK-GGKVKGKSKSRSSRAGLQFPVGRIHRMLRKGNYAERVGAGAPV    48

Hs H2A    50   YMAAVLEYLTAEILELAGNAARDNKKTRIIPRHLQLAIRNDEELNKLLGK    99
               |:|||||||.||:|||||||||||||||||||||||||||||||||||..
Rv H2A    49   YLAAVLEYLAAEVLELAGNAARDNKKTRIIPRHLQLAIRNDEELNKLLSG    98

Hs H2A   100   VTIAQGGVLPNIQAVLLPKK-----TESH----HKAKGK-----------   129
               ||||||||||||...|||||     |||.    .|:.||
Rv H2A    99   VTIAQGGVLPNIAPTLLPKKTSGGPTESEAAPTKKSGGKGGVKAEREGAQ   148

Hs H2A   130   ----   129

Rv H2A   149   SQEA   152
```

B

```
Hs H2B     0   MPEPAKSAPAPKKGSKKAVT--KAQK---KDGKKRKRSRKESYSVYVYKV    44
                      .|....|.||||.  ||||   ....|||:.||||||||:|||
Rv H2B     0   -------MPPKAGGGKKAVKSGKAQKAIRNPNDKRKKKRKESYSVYIYKV    42

Hs H2B    45   LKQVHPDTGISSKAMGIMNSFVNDIFERIAGEASRLAHYNKRSTITSREI    94
               |||||||||:|||||.||||||||||||||||||||:.|||:||||||||
Rv H2B    43   LKQVHPDTGVSSKAMAIMNSFVNDIFERIAGEASRLSQYNKKSTITSREI    92

Hs H2B    95   QTAVRLLLPGELAKHAVSEGTKAVTKYTSSK-   125
               |||||||||||||||||||||||||||||:.
Rv H2B    93   QTAVRLLLPGELAKHAVSEGTKAVTKYTSANK   124
```

C

```
Hs H3      0   MARTKQTARKSTGGKAPRKQLATKAARKSAPATGGVKKPHRYRPGTVALR    49
               ||||||||||||||||||||||||||||||||||||||||||||||||||
Rv H3      0   MARTKQTARKSTGGKAPRKQLATKAARKSAPATGGVKKPHRYRPGTVALR    49

Hs H3     50   EIRRYQKSTELLIRKLPFQRLVREIAQDFKTDLRFQSSAVMALQEASEAY    99
               ||||||||||||||||||||||||||||||||||||||||||||||:|||
Rv H3     50   EIRRYQKSTELLIRKLPFQRLVREIAQDFKTDLRFQSSAVMALQEAAEAY    99

Hs H3    100   LVGLFEDTNLCAIHAKRVTIMPKDIQLARRIRGERA   135
               ||||||||||||||||||||||||||||||||||||
Rv H3    100   LVGLFEDTNLCAIHAKRVTIMPKDIQLARRIRGERA   135
```

D

```
Hs H4      0   MSGRGKGGKGLGKGGAKRHRKVLRDNIQGITKPAIRRLARRGGVKRISGL    49
               |:||||||||||||||||||||||||||||||||||||||||||||||||
Rv H4      0   MTGRGKGGKGLGKGGAKRHRKVLRDNIQGITKPAIRRLARRGGVKRISGL    49

Hs H4     50   IYEETRGVLKVFLENVIRDAVTYTEHAKRKTVTAMDVVYALKRQGRTLYG    99
               ||||||||||||||||||||||||||||||||||||||||||||||||||
Rv H4     50   IYEETRGVLKVFLENVIRDAVTYTEHAKRKTVTAMDVVYALKRQGRTLYG    99

Hs H4    100   FGG   102
               ||.
Rv H4    100   FGL   102
```

Supplemental Figure S10. Sequence alignment of human and *R. varieornatus* core histone proteins. (*A–D*) The full-length sequences of human (Hs) and *R. varieornatus* (Rv) histones H2A, H2B, H3, and H4 were aligned using EMBOSS Needle (https://www.ebi.ac.uk/Tools/psa/-emboss_needle/). The alignments reveal that the core histones, including the H2A and H2B amino acid residues involved in interactions with Dsup (indicated in blue type), are conserved between humans and *R. varieornatus*. The aminoacid residues comprising the nucleosome acidic patch are underlined. The histone protein sequences from the *R. varieornatus* strain YOKOZUNA-1 (Hashimoto et al. 2016) were obtained using BLASTP (Altschul et al. 1997) against human histones, which were used to reconstitute nucleosomes in this study. To maintain consistency with the standard histone numbering nomenclature, the initiating methionine residues, which are typically removed from the histone proteins, are denoted as position zero ("0") in each sequence.

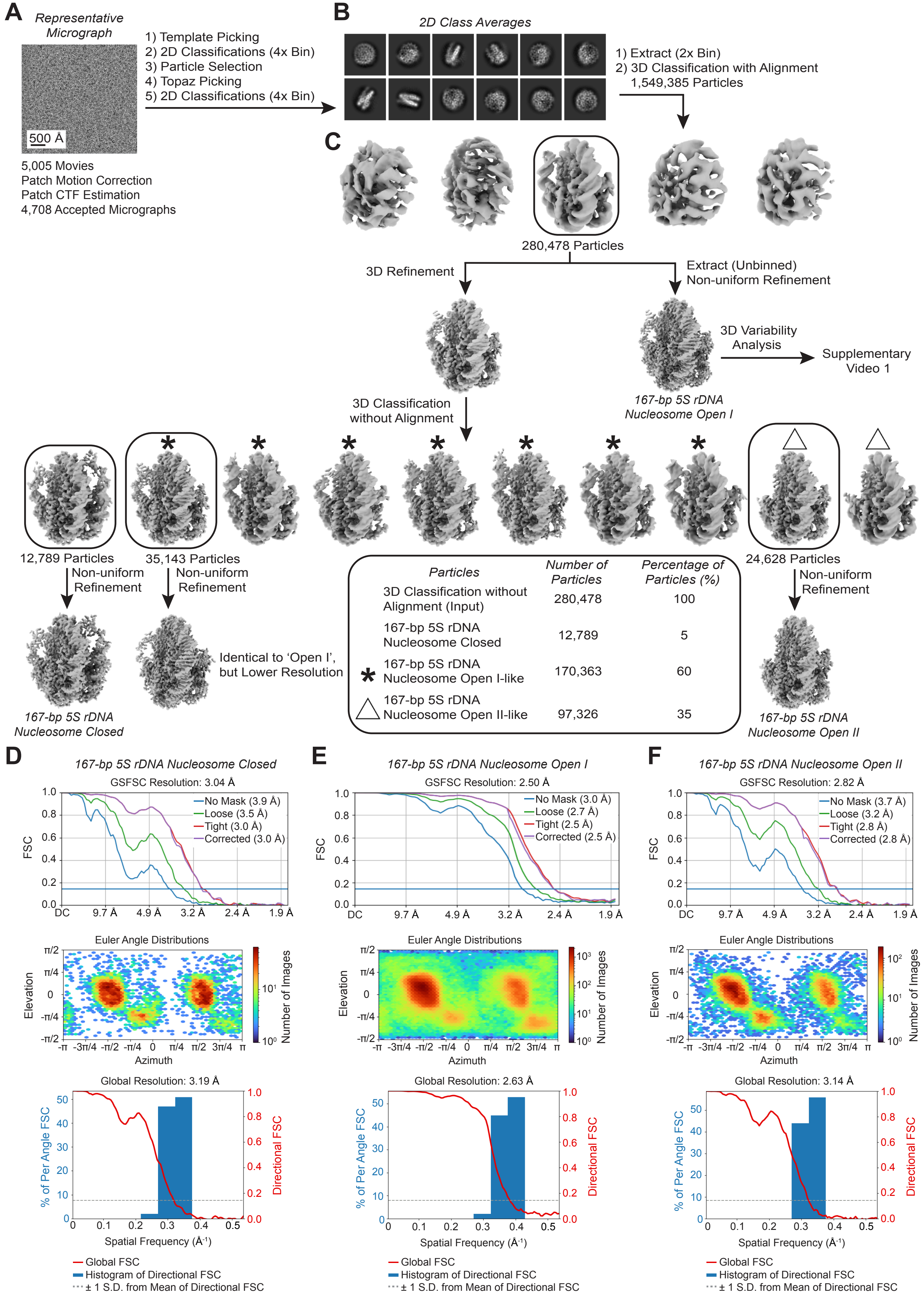

Alegrio-Louro et al. (Leschziner), Supplemental Fig. S11

Supplemental Figure S11. Image processing of the 167-bp 5S rDNA nucleosomes. (*A*) Representative micrograph, 10 Å low-pass filtered. (*B*) Selected 2D class averages. (*C*) 3D classifications and refinements. Classes marked with asterisks and triangles are 167-bp 5S rDNA nucleosome open I-like and open II-like, respectively. (*D–F*) Fourier shell correlations (FSC), particle angle distributions, and histogram of directional FSC for the 167-bp 5S rDNA nucleosome maps in the closed, open I, and open II conformations.

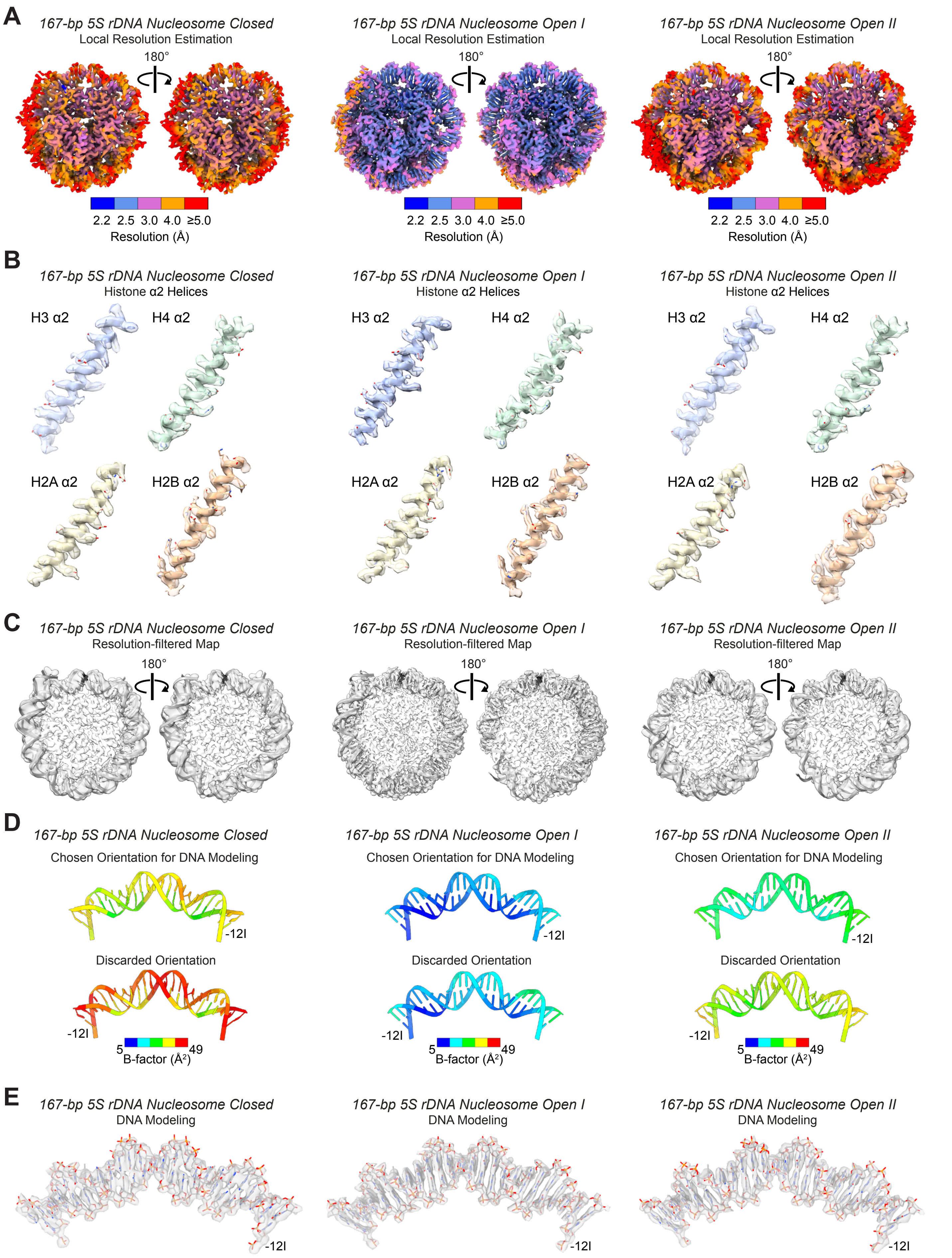

Alegrio-Louro et al. (Leschziner), Supplemental Fig. S12

Supplemental Figure S12. Local resolution estimation, map features, and DNA orientation analysis of the 167-bp 5S rDNA nucleosome structures. (*A*) Local resolution estimations plotted onto the maps. (*B*) Cryo-EM densities and models of the α2 helices of the histones in the three 167-bp 5S rDNA nucleosome states. (*C*) Maps filtered according to the local resolution estimation. The modeled DNA is shown as a ribbon. Histone models are removed from the map for clarity. We built a model for the DNA whenever density was observed in a map that had been filtered according to the estimated local resolution, without lowering the default display contour levels in ChimeraX (Goddard et al. 2018). (*D*) DNA orientation analysis. To distinguish between the two possible orientations of the modeled DNA (related through a 180° rotation along the dyad axis), we refined the 25 bp of nucleosomal DNA centered around the dyad (position 0) in those two orientations. The 25 bp are colored by the per-bp B-factors (see Supplemental Materials and methods). Lower B-factors indicate higher certainty about the atomic 3D coordinates. For the three structures, the B-factors are consistently lower for the same orientation. (*E*) Cryo-EM densities and chosen models of the 25-bp DNA centered around the dyad. We chose this DNA segment because of its high-resolution features in the three 167-bp 5S rDNA nucleosome maps.

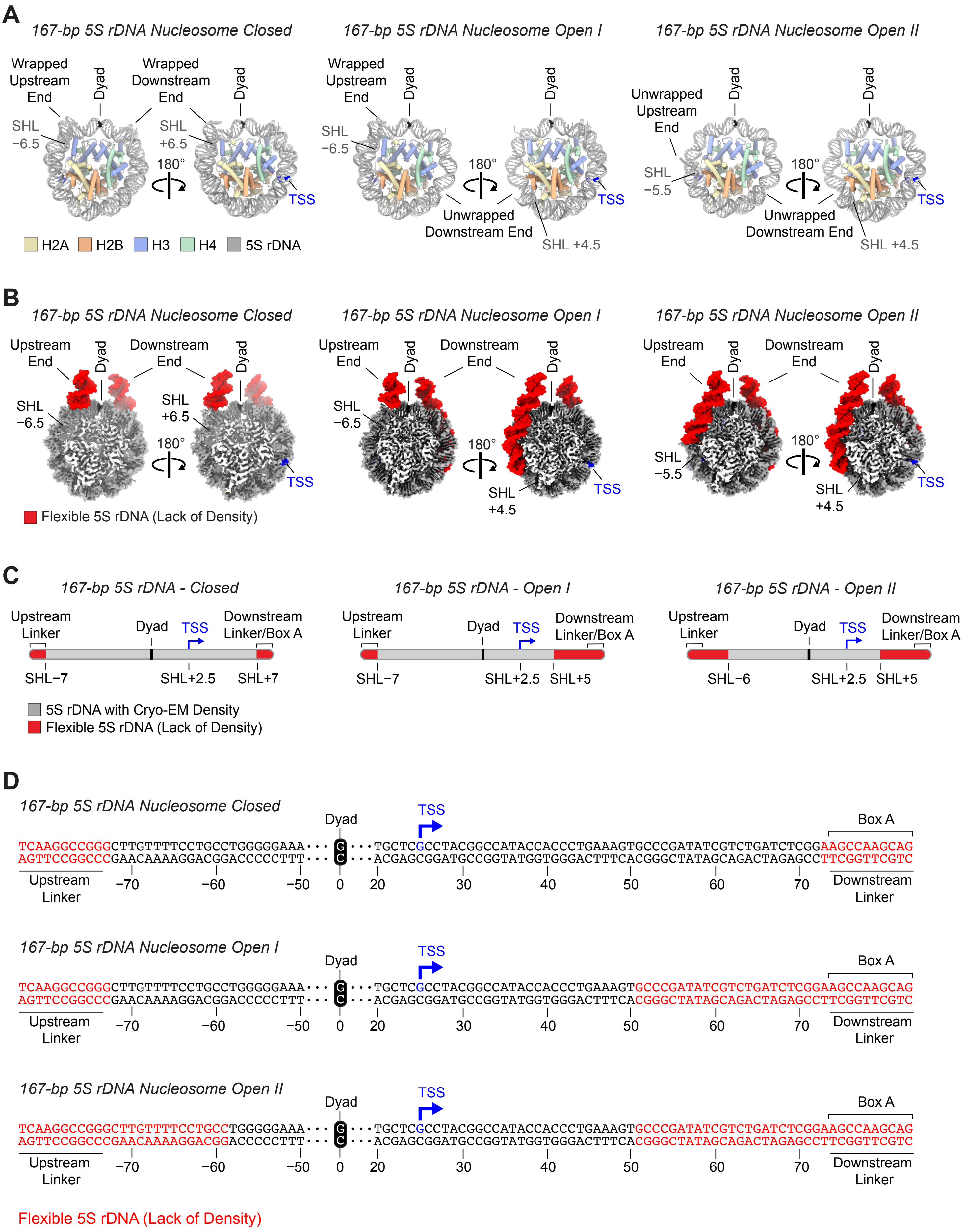

Alegrio-Louro et al. (Leschziner), Supplemental Fig. S13

Supplemental Figure S13. The DNA end that is downstream of the 5S rDNA TSS is preferentially unwrapped in the 167-bp nucleosome. (*A*) Models of the 167-bp 5S rDNA nucleosome in the closed, open I, and open II conformations. (*B*) Cryo-EM maps with the addition of molecular models, in red, to indicate the DNA segments that lack cryo-EM density (flexible DNA). SHL: superhelical location. (*C*) Schematics of the 167-bp 5S rDNA fragment with the regions that are absent in the cryo-EM maps of the closed, open II, and open II nucleosome conformations represented in red. The DNA ends are labeled as upstream or downstream relative to the transcription start site (TSS). SHL: superhelical location. (*D*) Flexible DNA sequence in the 167-bp 5S rDNA nucleosomes. The DNA sequence in red type is not visible in the cryo-EM maps. The downstream linker sequence (AGCCAAGCAG) corresponds to most of the Box A sequence (AGCCAAGCAGGG) in the promoter of 5S rRNA genes (Pieler et al. 1987). The numbers indicate the bp position relative to the dyad. The full 167-bp 5S rDNA sequence is available in the Supplemental Materials and methods.

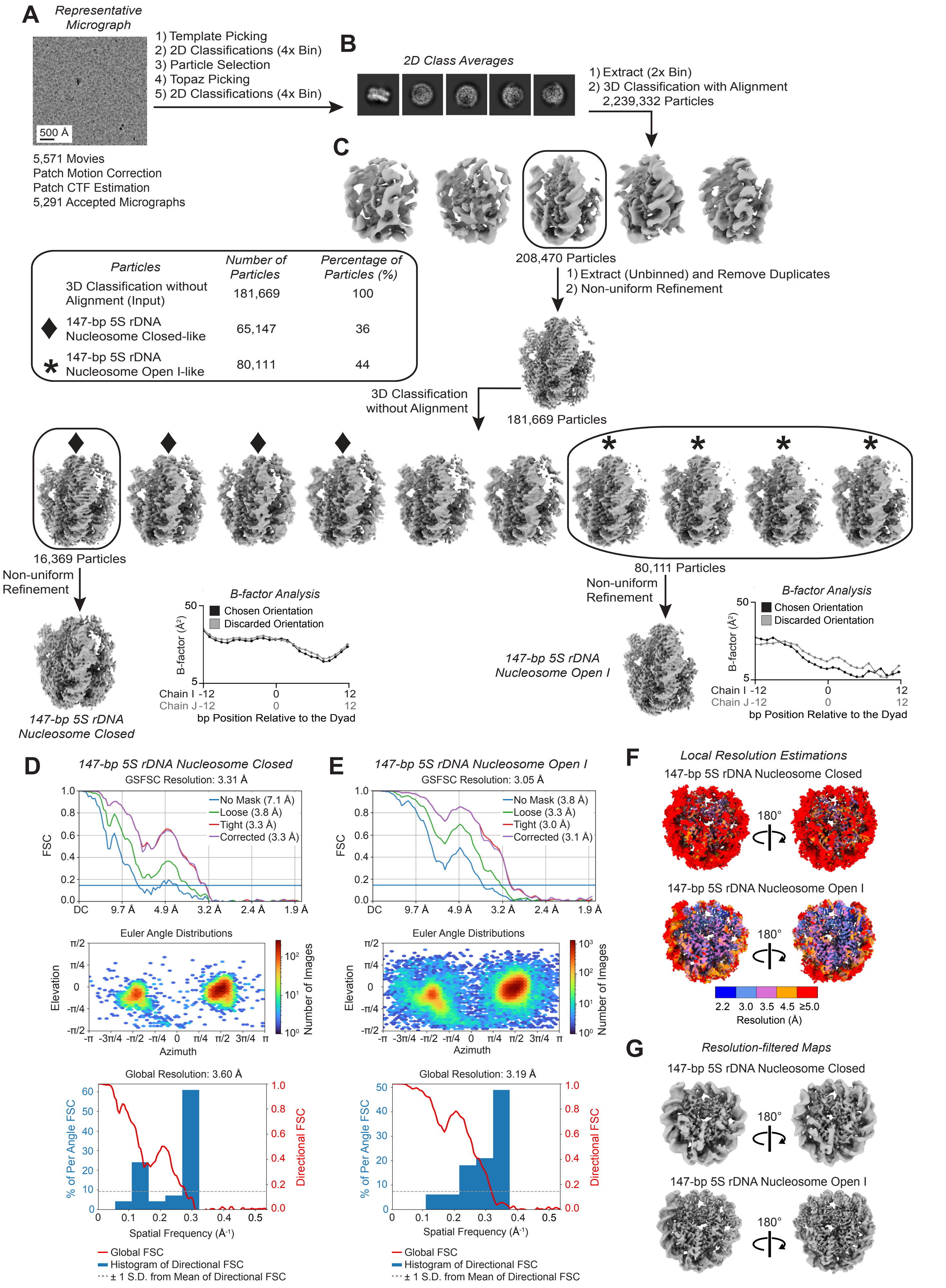

Alegrio-Louro et al. (Leschziner), Supplemental Fig. S14

Supplemental Figure S14. Image processing of the 147-bp 5S rDNA nucleosomes. (*A*) Representative aligned micrograph, 10 Å low-pass filtered. (*B*) Selected 2D class averages. (*C*) 3D classifications and refinements. Classes marked with diamonds and asterisks are 147-bp 5S rDNA nucleosome closed-like and open I-like, respectively. (*D–E*) Fourier shell correlations (FSC), particle angle distributions, and histogram of directional FSC for the 147-bp 5S rDNA nucleosome maps. (*F*) Local resolution estimations plotted onto the maps. (*G*) Maps filtered according to the local resolution estimation.

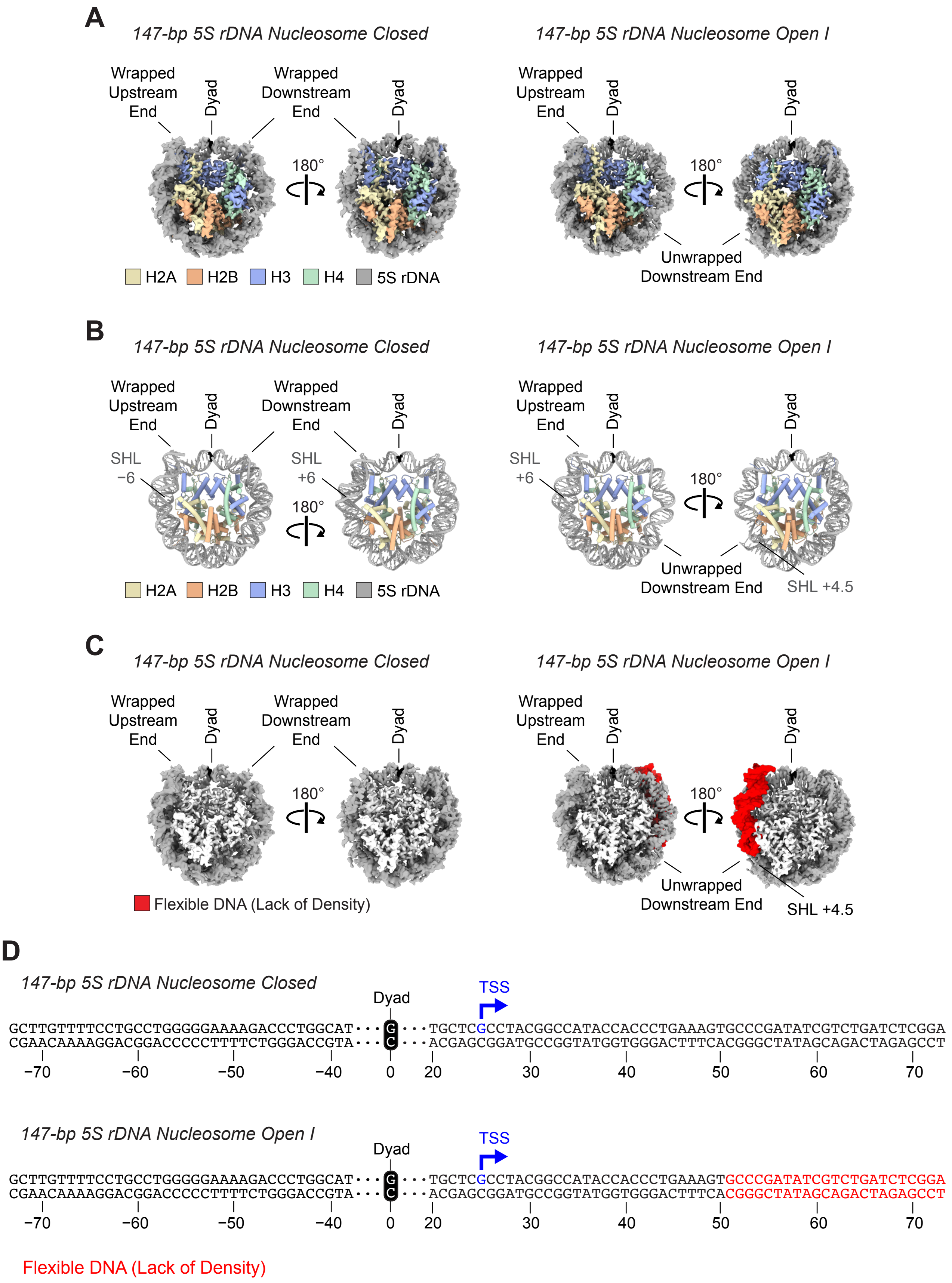

A
147-bp 5S rDNA Nucleosome Closed
Wrapped Upstream End
Dyad
Wrapped Downstream End
Dyad
180°
H2A
H2B
H3
H4
5S rDNA
147-bp 5S rDNA Nucleosome Open I
Wrapped Upstream End
Dyad
Dyad
180°
Unwrapped Downstream End
B
147-bp 5S rDNA Nucleosome Closed
Wrapped Upstream End
Dyad
Wrapped Downstream End
Dyad
SHL −6
SHL +6
180°
H2A
H2B
H3
H4
5S rDNA
147-bp 5S rDNA Nucleosome Open I
Wrapped Upstream End
Dyad
Dyad
SHL +6
180°
Unwrapped Downstream End
SHL +4.5
C
147-bp 5S rDNA Nucleosome Closed
Wrapped Upstream End
Dyad
Wrapped Downstream End
Dyad
180°
Flexible DNA (Lack of Density)
147-bp 5S rDNA Nucleosome Open I
Wrapped Upstream End
Dyad
Dyad
180°
Unwrapped Downstream End
SHL +4.5
D
147-bp 5S rDNA Nucleosome Closed
Dyad
TSS
GCTTGTTTTCCTGCCTGGGGGAAAAGACCCTGGCAT···G···TGCTCGCCTACGGCCATACCACCCTGAAAGTGCCCGATATCGTCTGATCTCGGA
CGAACAAAAGGACGGACCCCCTTTTCTGGGACCGTA···C···ACGAGCGGATGCCGGTATGGTGGGACTTTCACGGGCTATAGCAGACTAGAGCCT
−70 −60 −50 −40 0 20 30 40 50 60 70
147-bp 5S rDNA Nucleosome Open I
Dyad
TSS
GCTTGTTTTCCTGCCTGGGGGAAAAGACCCTGGCAT···G···TGCTCGCCTACGGCCATACCACCCTGAAAGTGCCCGATATCGTCTGATCTCGGA
CGAACAAAAGGACGGACCCCCTTTTCTGGGACCGTA···C···ACGAGCGGATGCCGGTATGGTGGGACTTTCACGGGCTATAGCAGACTAGAGCCT
−70 −60 −50 −40 0 20 30 40 50 60 70
Flexible DNA (Lack of Density)

Supplemental Figure S15. The 147-bp 5S rDNA nucleosome exists in a closed form as well as in an open form that is unwrapped downstream of the TSS. (*A*) Cryo-EM maps of the 147-bp 5S rDNA nucleosome in the closed (both DNA ends wrapped) and open I (one wrapped and one unwrapped DNA end) conformations. (*B*) Models built on the cryo-EM maps of the 147-bp 5S rDNA nucleosome structures. SHL: superhelical location. (*C*) Cryo-EM maps with the addition of red molecular models, which indicate the DNA segments that lack cryo-EM density. (*D*) Flexible DNA sequence in the 147-bp 5S rDNA nucleosome. The DNA sequence in red type is not visible in the cryo-EM map of the 147-bp 5S rDNA nucleosome open I conformation. The numbers indicate the bp position relative to the dyad. The 5S rDNA ends in panels *A*–*C* are labeled as upstream or downstream relative to the transcription start site (TSS). The full 147-bp 5S rDNA sequence is available in the Supplemental Materials and methods.

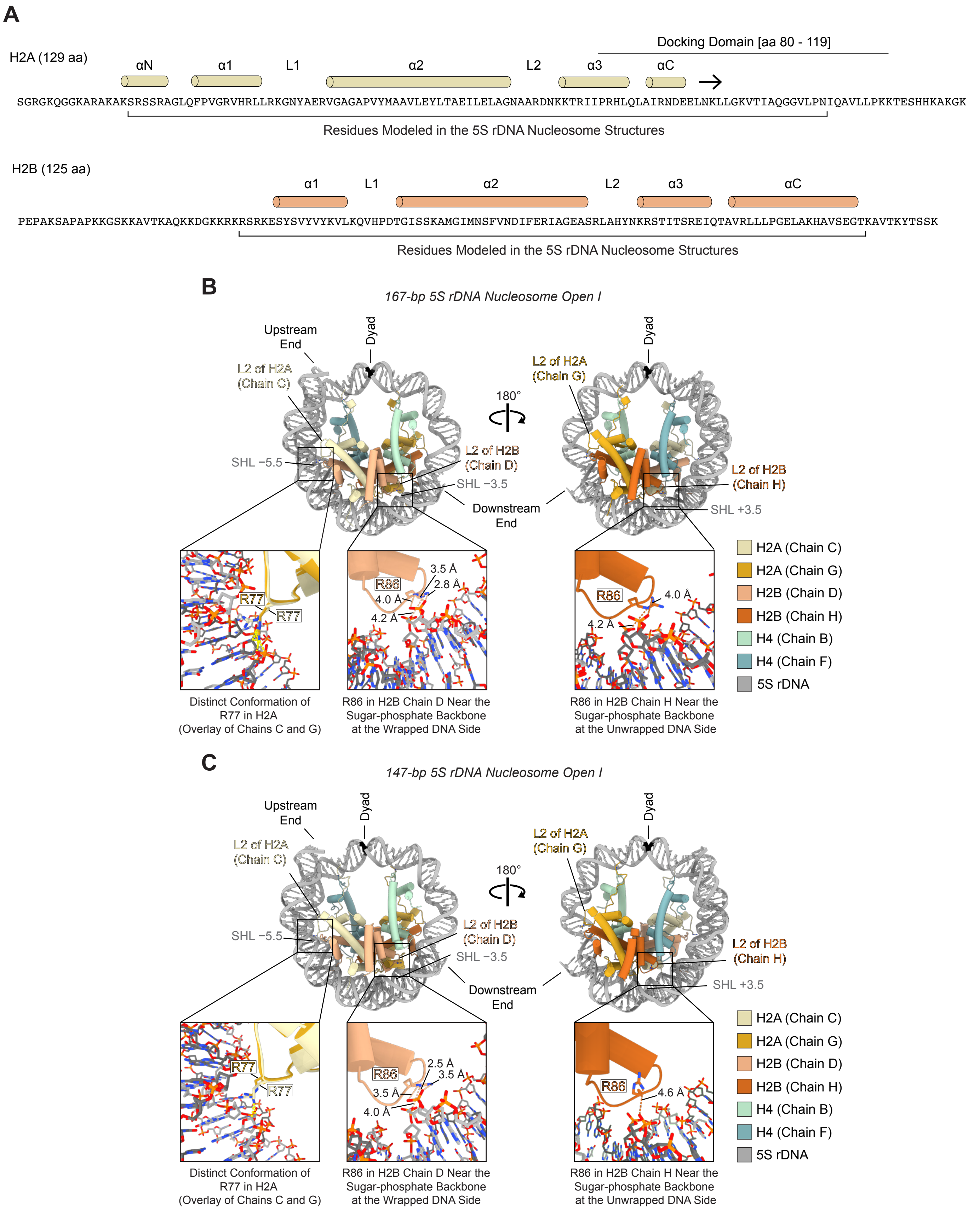

Alegrio-Louro et al. (Leschziner), Supplemental Fig. S16

Supplemental Figure S16. Distinct conformations of H2A R77 and H2B R86 in their interactions with wrapped versus unwrapped 5S rDNA in the nucleosome. (*A*) Sequence and secondary structure of H2A and H2B. α-helices and β-strands are represented by cylinders and arrows. L: Loop; N: N-terminal; C: C-terminal. The brackets highlight the histone sequence that is modeled in the structures of the 167-bp and the 147-bp 5S rDNA nucleosomes. (*B*,*C*) Models of the 167-bp and the 147-bp 5S rDNA nucleosomes in the open I conformation (one wrapped DNA end and one unwrapped DNA end) with close-up views of H2A R77 and H2B R86 on the two faces of the nucleosomes. Histone H3 is removed from the models for clarity. In the close-up images on the left, H2A (chain C) near the upstream DNA end is overlaid onto H2A (chain G) near the downstream DNA end to show that H2A R77 facing the flexible downstream end would clash (yellow dashed lines) with a fully wrapped outer DNA turn. The close-up views of H2B R86 on each of the two nucleosome faces (middle and right inset images) depict anticipated electrostatic interactions with the sugar-phosphate backbone of the DNA. The numbers indicate the distance (in Å) between H2B R86 and the sugar phosphate backbone. SHL: superhelical location.

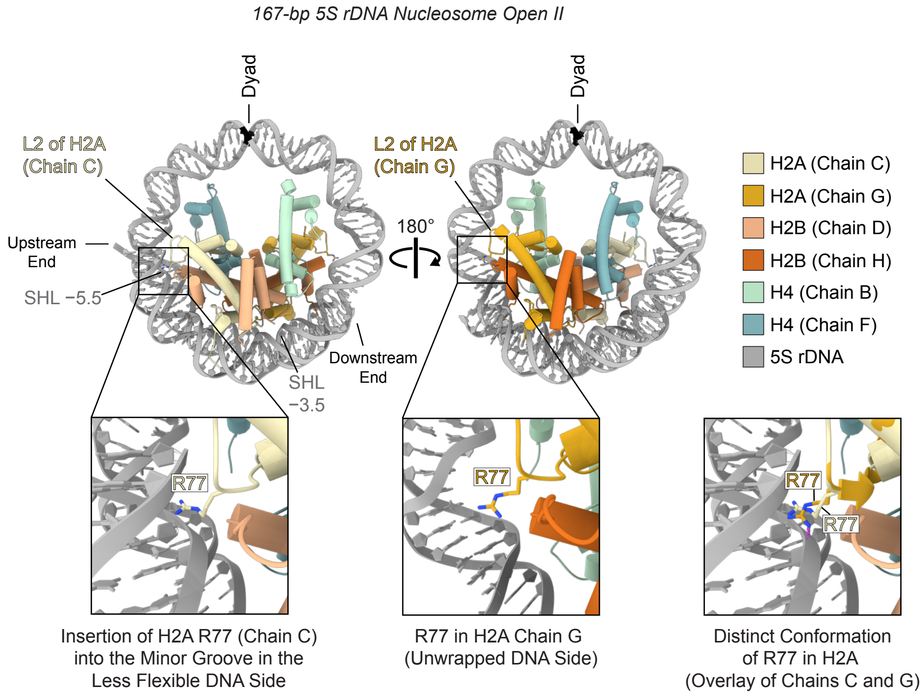

Supplemental Figure S17. H2A R77 inserts into the minor groove in one side of the 167-bp 5S rDNA nucleosome in the open II conformation. Model with close-up views of H2A R77 on each of the two nucleosome faces. Histone H3 is removed from the model for clarity. R77 of H2A chain C interacts with the 5S rDNA near to the SHL –5.5 at the less flexible DNA side. In contrast, R77 of H2A chain G (near the most flexible downstream DNA side) would collide (depicted by purple dashed lines in the inset image on the right) with DNA wrapped at the SHL +5.5. SHL: superhelical location.

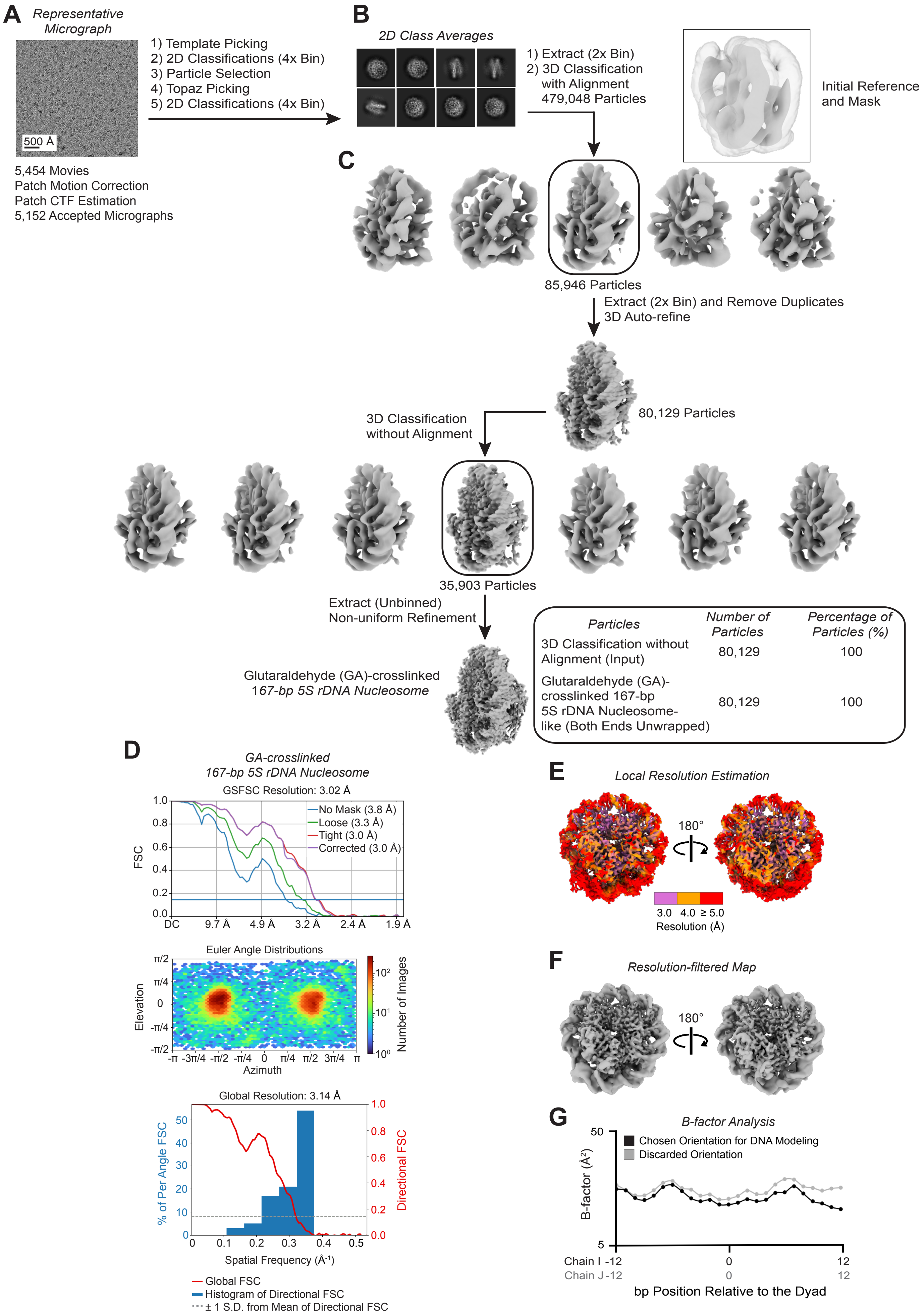

Alegrio-Louro et al. (Leschziner), Supplemental Fig. S18

Supplemental Figure S18. Image processing of the glutaraldehyde-crosslinked 167-bp 5S rDNA nucleosomes. (*A*) Representative micrograph, 10 Å low-pass filtered. (*B*) Selected 2D class averages. (*C*) 3D classifications and refinements. Note that the initial reference for 3D classifications with alignment presented one DNA end wrapped around the octamer but the 3D classification output volume displayed both ends flexible. (*D*) Fourier shell correlation (FSC), particle angle distribution, and histogram of directional FSC. (*E*) Local resolution estimation plotted onto the map. (*F*) Map filtered according to the local resolution estimation. (*G*) Average per-bp B factors plotted for the 25 bp refined in the two possible orientations.

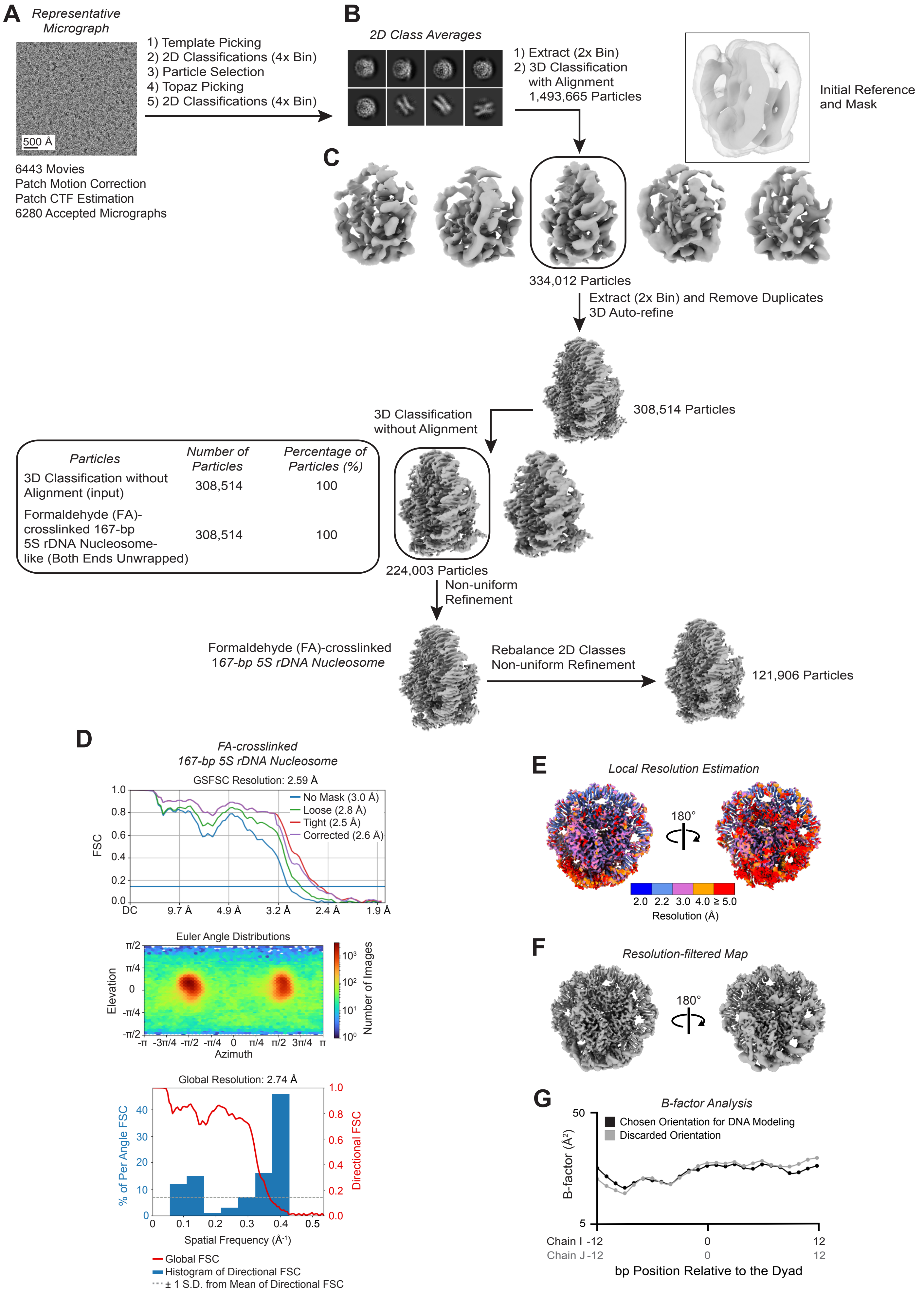

Alegrio-Louro et al. (Leschziner), Supplemental Fig. S19

Supplemental Figure S19. Image processing of the formaldehyde-crosslinked 167-bp 5S rDNA nucleosomes. (*A*) Representative micrograph, 10 Å low-pass filtered. (*B*) Selected 2D class averages. (*C*) 3D classifications and refinements. Note that the initial reference for 3D classifications with alignment presented one DNA end wrapped around the octamer but the 3D classification output volume displayed both ends flexible. (*D*) Fourier shell correlation (FSC), particle angle distribution, and histogram of directional FSC. (*E*) Local resolution estimation plotted onto the map. (*F*) Map filtered according to the local resolution estimation. (*G*) Average per-bp B factors plotted for the 25 bp refined in the two possible orientations.

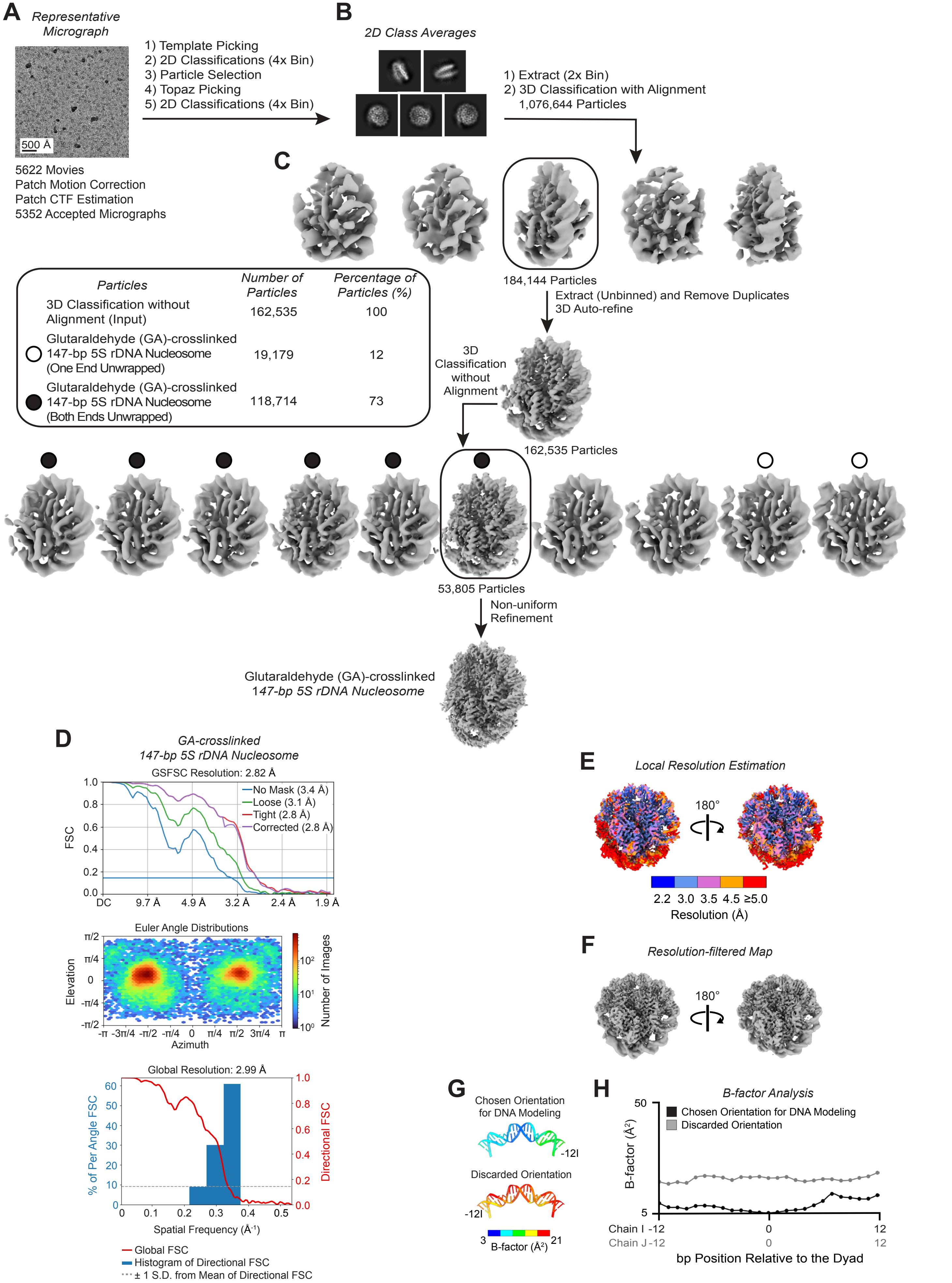

Alegrio-Louro et al. (Leschziner), Supplemental Fig. S20

Supplemental Figure S20. Image processing of the glutaraldehyde-crosslinked 147-bp 5S rDNA nucleosomes. (*A*) Representative micrograph, 10 Å low-pass filtered. (*B*) Selected 2D class averages. (*C*) 3D classifications and refinements. (*D*) Fourier shell correlation (FSC), particle angle distribution, and histogram of directional FSC. (*E*) Local resolution estimation plotted onto the map. (*F*) Map filtered according to the local resolution estimation. (*G*) DNA, real-space refined into the map, colored according to the per-bp B-factors (see Supplemental Materials and methods). Only the 25 bp of nucleosomal DNA centered on the dyad, which is the region of highest local resolution, are shown here. The DNA was refined in the two possible orientations (related through a 180° rotation along the dyad axis). We used the orientation with the lower B-factors to build the final atomic models. (*H*) Average per-bp B factors plotted for the 25 bp refined in the two possible orientations.

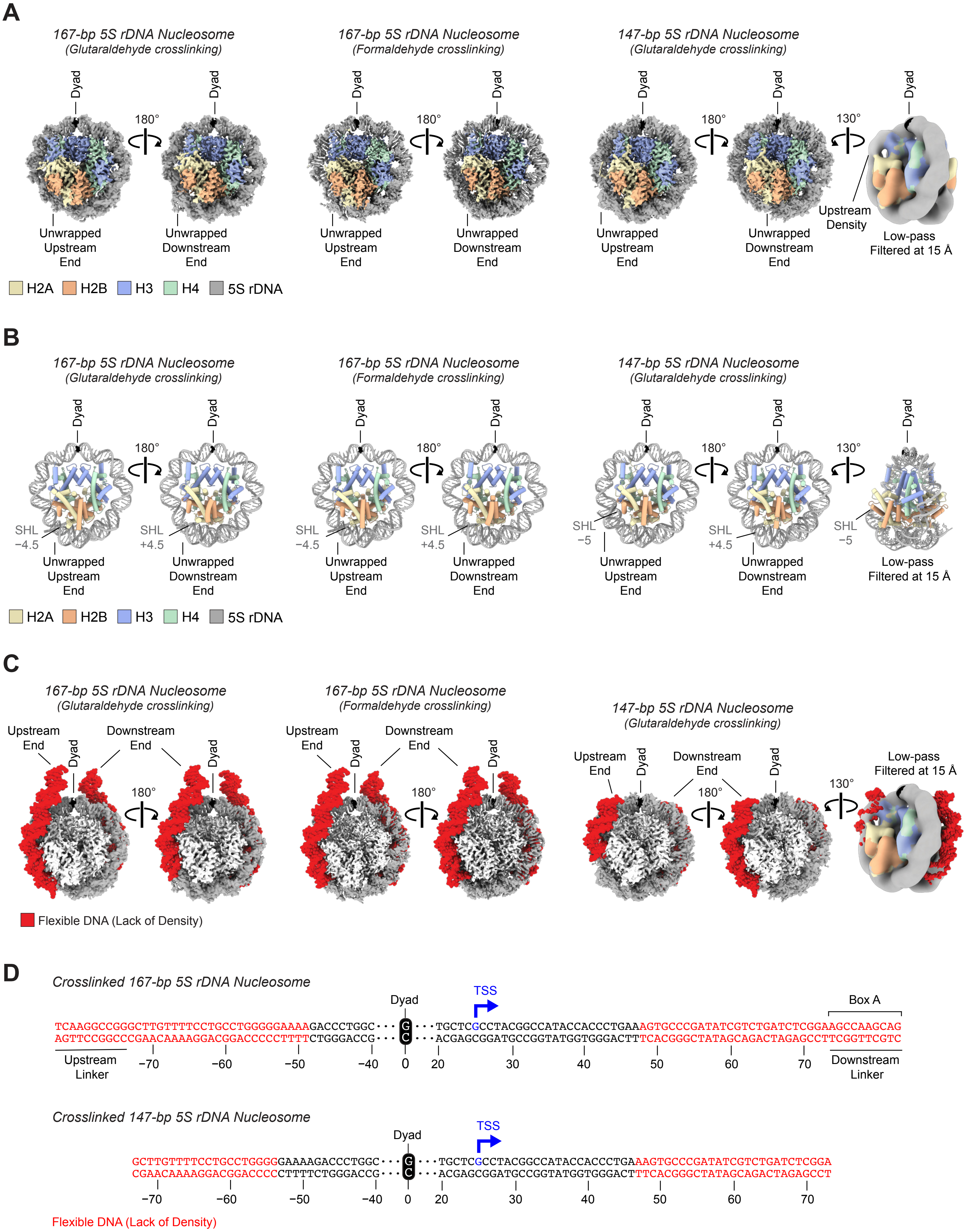

Alegrio-Louro et al. (Leschziner), Supplemental Fig. S21

Supplemental Figure S21. Crosslinking of the 167-bp and 147-bp 5S rDNA nucleosomes results in DNA unwrapping. (*A*) Cryo-EM density maps of the 5S rDNA nucleosomes cross-linked with either glutaraldehyde or formaldehyde. (*B*) Models of the crosslinked 5S rDNA nucleosomes. SHL: superhelical location. (*C*) Cryo-EM maps with the addition of red molecular models, which indicate the DNA segments that lack density (flexible DNA) in the crosslinked 5S rDNA nucleosomes. (*D*) Flexible DNA sequence in the crosslinked 5S rDNA nucleosomes. The DNA sequence in red type is not visible in the cryo-EM maps. The numbers indicate the bp position relative to the dyad. The 5S rDNA ends in panels *A–C* are labeled as upstream or down-stream relative to the transcription start site (TSS). A low pass-filtered version of the maps (farthest right images in panels *A–C*) highlights density that is poorly resolved in the unfiltered map of the 147-bp 5S rDNA nucleosome. The full 167-bp and 147-bp 5S rDNA sequences are available in the Supplemental Materials and methods.

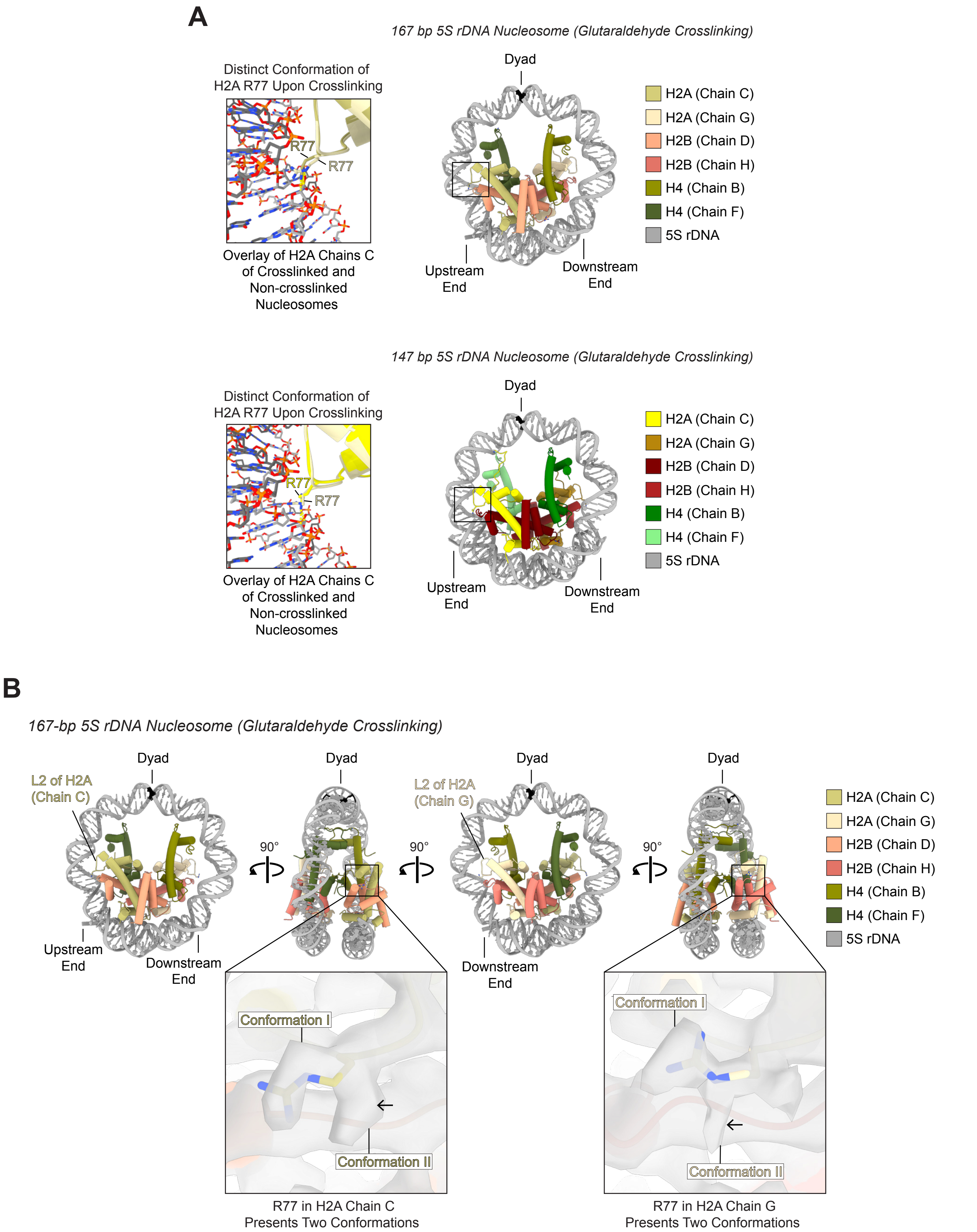
A
167 bp 5S rDNA Nucleosome (Glutaraldehyde Crosslinking)
Distinct Conformation of H2A R77 Upon Crosslinking
R77
R77
Overlay of H2A Chains C of Crosslinked and Non-crosslinked Nucleosomes
Dyad
Upstream End
Downstream End
H2A (Chain C)
H2A (Chain G)
H2B (Chain D)
H2B (Chain H)
H4 (Chain B)
H4 (Chain F)
5S rDNA
147 bp 5S rDNA Nucleosome (Glutaraldehyde Crosslinking)
Distinct Conformation of H2A R77 Upon Crosslinking
R77
R77
Overlay of H2A Chains C of Crosslinked and Non-crosslinked Nucleosomes
Dyad
Upstream End
Downstream End
H2A (Chain C)
H2A (Chain G)
H2B (Chain D)
H2B (Chain H)
H4 (Chain B)
H4 (Chain F)
5S rDNA
B
167-bp 5S rDNA Nucleosome (Glutaraldehyde Crosslinking)
Dyad
L2 of H2A (Chain C)
Upstream End
Downstream End
90°
Dyad
90°
Dyad
L2 of H2A (Chain G)
Downstream End
90°
Dyad
H2A (Chain C)
H2A (Chain G)
H2B (Chain D)
H2B (Chain H)
H4 (Chain B)
H4 (Chain F)
5S rDNA
Conformation I
Conformation II
R77 in H2A Chain C Presents Two Conformations
Conformation I
Conformation II
R77 in H2A Chain G Presents Two Conformations

Supplemental Figure S22. H2A R77 adopts two different conformations on each side of the glutaraldehyde-crosslinked 167-bp 5S rDNA nucleosome. (*A*) Atomic models of the 167-bp and 147-bp 5S rDNA nucleosomes crosslinked with glutaraldehyde with close-up views of H2A R77. In the images on the left, the H2A chains C of crosslinked and non-crosslinked nucleosomes are overlaid to show that upon crosslinking H2A R77 adopts a conformation that would collide (depicted by yellow dashed lines) with a fully wrapped outer DNA turn. The H2A chains C of the non-crosslinked nucleosomes are the same as in Supplemental Fig. S15. Histone H3 is removed from the model for clarity. (*B*) The close-up views of the 167-bp 5S rDNA nucleosome cross-linked with glutaraldehyde show the cryo-EM density, in light gray, for the two conformations of H2A R77 on each of the two nucleosome sides, as well as the modeled H2A R77 (conformation I). The arrows indicate the unmodeled conformation (conformation II) of R77 in H2A chains C and G.

A

Cα (Histones) and C4' (DNA) Root-mean-square Deviation (RMSD)
167-bp 5S rDNA Nucleosome Closed vs. 167-bp 5S rDNA Nucleosome Open I

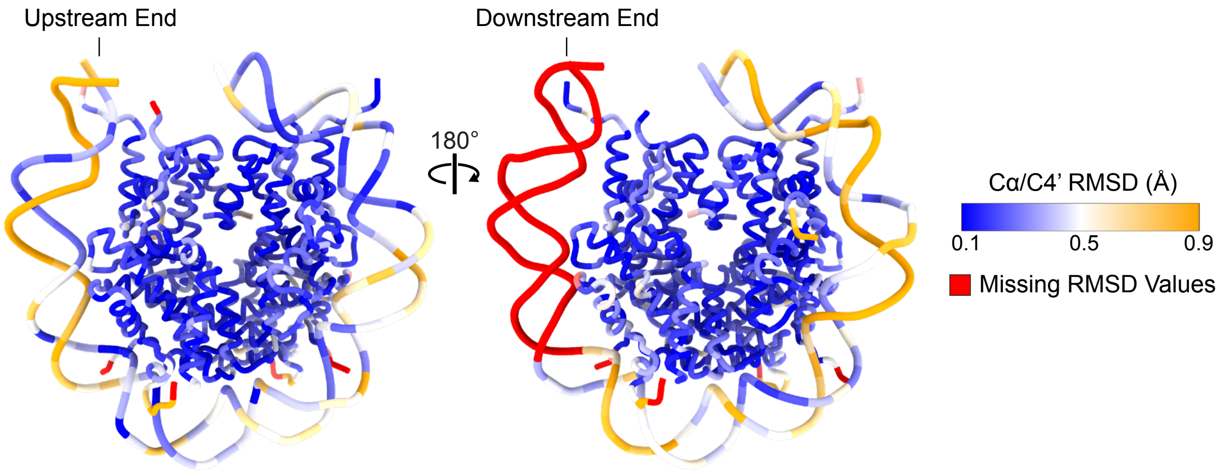

B

Cα (Histones) and C4' (DNA) Root-mean-square Deviation (RMSD)
167-bp 5S rDNA Nucleosome Closed vs. 167-bp 5S rDNA Nucleosome Open II

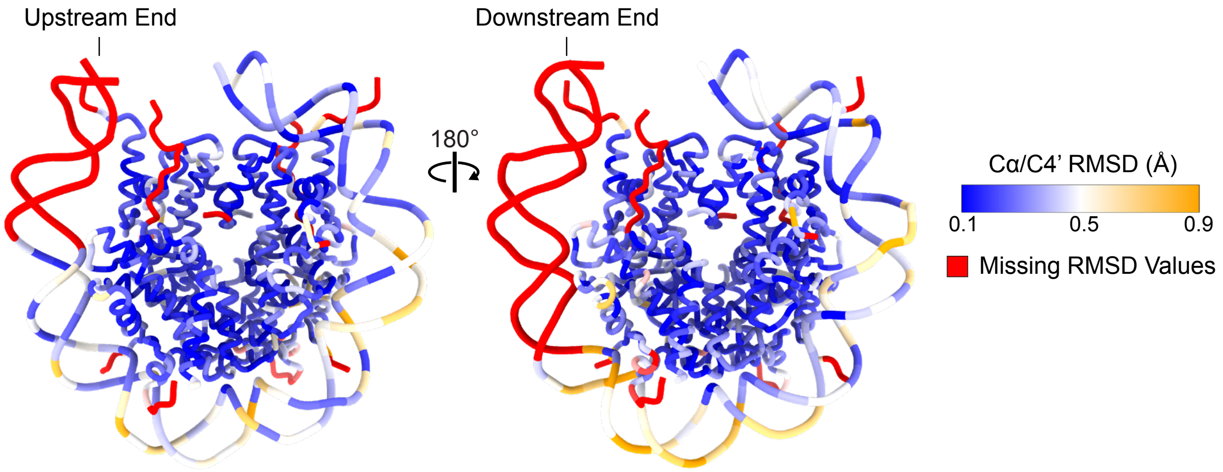

C

Cα (Histones) and C4' (DNA) Root-mean-square Deviation (RMSD)
167-bp 5S rDNA Nucleosome Closed vs. Glutaraldehyde (GA)-crosslinked 167-bp 5S rDNA Nucleosome

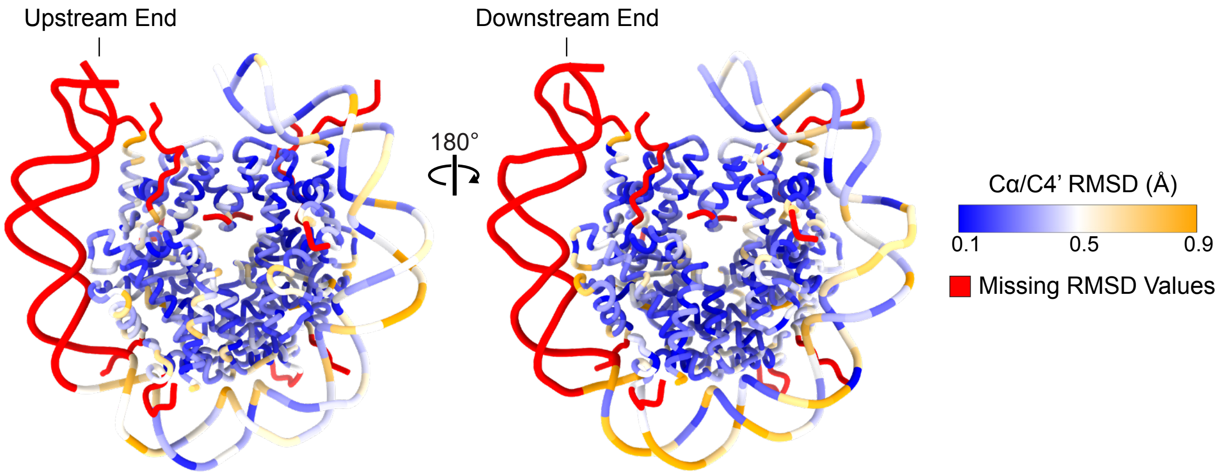

Supplemental Figure S23. Root-mean-square deviation (RMSD) of Cα atoms (histones) and C4' atoms (DNA) between the models of 167-bp 5S rDNA Nucleosome: Closed, Open I, Open II and Glutaraldehyde (GA)-crosslinked. All RMSD values are calculated relative to the 167-bp 5S rDNA Nucleosome Closed model. Values that could not be determined due to lack of atomic coordinates in one of the models are represented in red.

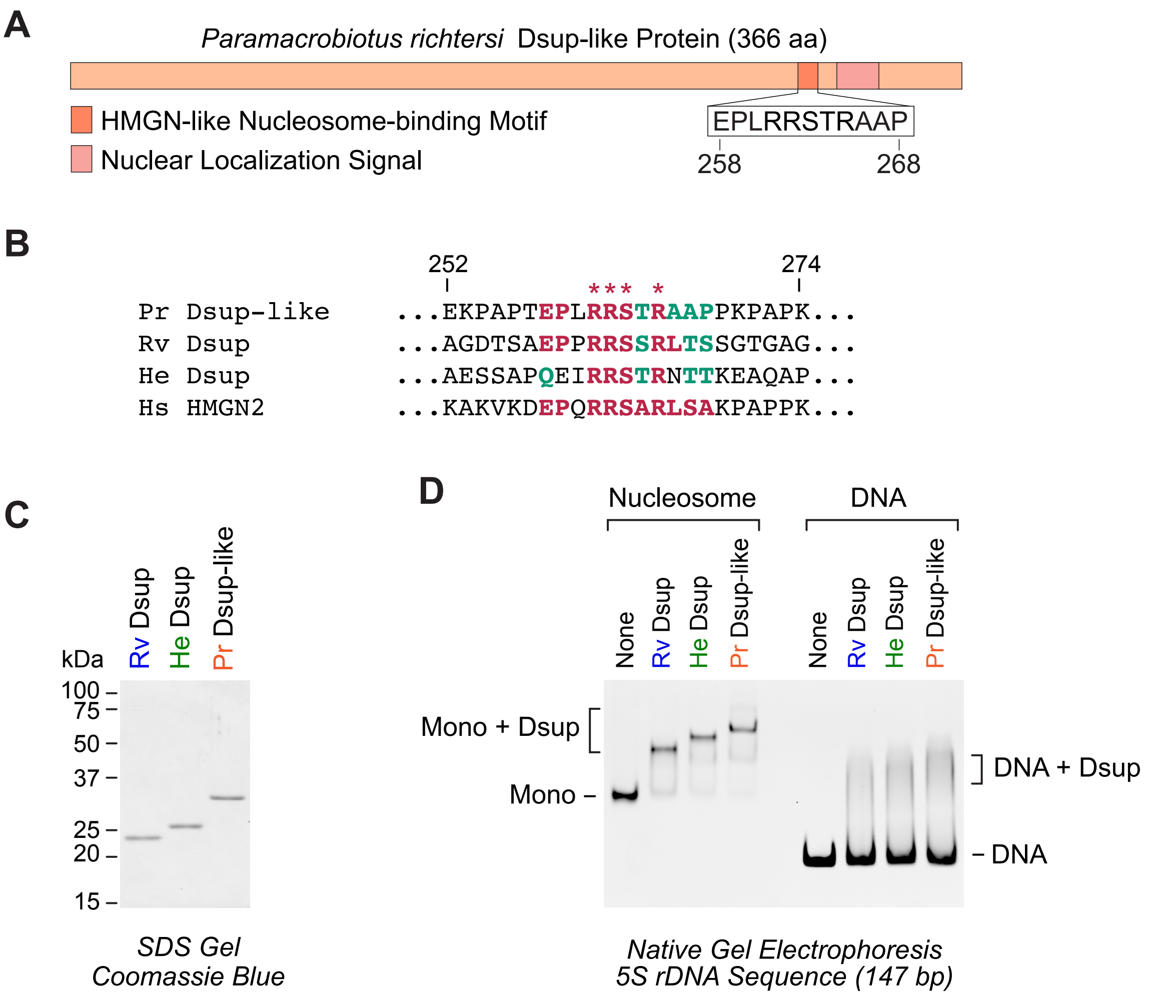

Supplemental Figure S24. The HMGN motif is conserved across multiple tardigrade species. (*A*) *P. richtersi* Dsup-like protein. The HMGN-like nucleosome-binding motif is highlighted (residues 258-268). (*B*) Alignment of Dsup proteins from the tardigrades *R. varieornatus* (Rv), *H. exemplaris* (He), and *P. richtersi* (Pr) with the human (Hs) HMGN2 protein as a representative member of the HMGN protein family. Partial sequences of Rv Dsup, He Dsup, Pr Dsup-like and Hs HMGN2 proteins are shown. Identical amino acid residues are highlighted in red type, and conserved amino acid substitutions (as in, for example, Wu and Brutlag, 1996) are in green type. The numbers indicate the amino acid residues in Pr Dsup. The asterisks denote the Rv Dsup residues that interact with the acidic patch. (*C*) Purification of recombinant Rv Dsup (residues 306-445), He Dsup (residues 183-328), and Pr Dsup-like (residues 182-366) C-terminal protein regions containing an HMGN motif. The recombinant proteins have an N-terminal His6-tag and a C-terminal FLAG tag. The purified proteins were analyzed by 12% polyacrylamide-SDS gel electrophoresis and staining with Coomassie Brilliant Blue R-250. (*D*) The Pr Dsup-like protein binds to nucleosomes. Gel mobility shift experiments were performed as described in Chavez et al. (2019) with the 5S rDNA sequence in the form of free DNA or mononucleosomes (mono) with purified recombinant Dsup proteins as noted. The positions of mononucleosomes, free DNA fragments (DNA), Dsup-nucleosome complexes (Mono + Dsup), and Dsup-DNA complexes (DNA + Dsup) are indicated.

Sample	**147-bp 601 nucleosome crosslinked to Dsup**		**167-bp 5S rDNA nucleosome crosslinked to HMGN2**	**167-bp 5S rDNA nucleosome crosslinked to HMGN5**
Structures	Dsup-bound Nucleosome Structure I	Dsup-bound Nucleosome Structure II		
Accession codes	PDB 9D3L<br>EMD-46537	PDB 9D3K<br>EMD-46536	PDB 9D3M<br>EMD-46538	EMD-46539
Type of support	Quantifoil R2/1, UT, 300 Mesh, Copper (Cat No. Q350CR1-2nm)			
Microscope	Titan Krios G4 (Thermo Fisher Scientific)			
Detector	Falcon 4			
Energy filter	Selectris X			
Acceleration voltage (kV)	300			
Pixel size (Å)	0.935			
Total dose (e$^-$/Å^2)	50			
No. of micrographs recorded/used	10949/10056		13833/11761	13648/12318
Symmetry	C1 (symmetry not imposed)			
No. of picked particles	3,767,974 (100%)		4,738,442 (100%)	2,166,837 (100%)
No. of particles after 2D classification	644,478 (17%)		1,079,681 (50%)	2,279,409 (48%)
No. of particles after 3D classification with alignment	N/A		55,942 (3%)	220,875 (5%)
No. of particles in the final reconstruction	194,251 (5%)	371,055 (10%)	55,942 (3%)	220,875 (5%)
Global resolution (Å)	2.8	2.7	2.9	2.9
**Refinement**				
Model resolution (Å) (FSC threshold 0.143)	2.8	2.7	2.9	N/A
**Model composition**				
Chains	12	12	12	
Non-hydrogen atoms	10994	9677	10203	
Protein residues	752	742	765	
Nucleotide	248	188	202	
**Mean B-factors (Å^2)**				
Protein	48.22	30.35	63.46	
Nucleotide	99.18	61.59	130.20	
**R.m.s. deviations**				
Bond lengths (Å)	0.004	0.004	0.005	
Bond angles (°)	0.659	0.654	0.539	
**Validation**				
MolProbity score	1.77	1.84	1.60	
Clash score	7.52	8.03	6.53	
**Ramachandran plot**				
Outliers (%)	0.00	0.14	0.00	
Allowed (%)	1.50	2.49	2.28	
Favored (%)	98.50	97.37	97.72	

Sample	**167-bp 5S rDNA nucleosome**		
Structures	167-bp 5S rDNA Nucleosome Closed	167-bp 5S rDNA Nucleosome Open I	167-bp 5S rDNA Nucleosome Open II

Accession codes	EMD - 46542<br>PDB 9D3O	PDB 9D3P<br>EMD-46543	PDB 9D3Q<br>EMD-46544
Type of support	Quantifoil R2/1, UT, 300 Mesh, Copper (Cat No. Q350CR1-2nm)		
Microscope	Titan Krios G4 (Thermo Fisher Scientific)		
Detector	Falcon 4		
Energy filter	Selectris X		
Acceleration voltage (kV)	300		
Pixel size (Å)	0.935		
Total dose (e^-/Å^2)	50		
No. of micrographs recorded/used	5005/4708		
Symmetry	C1 (symmetry not imposed)		
No. of picked particles	2,263,256 (100%)		
No. of particles after 2D classifications	1,549,385 (68%)		
No. of particles after 3D classification with alignment	280,478 (12%)		
No. of particles in the final reconstruction after 3D classification without alignment	12,789 (0.6%)	280,478 (12%)	24,628 (1%)
Global resolution (Å)	3.0	2.5	2.8
**Refinement**			
Model resolution (Å) (FSC threshold 0.143)	3.0	2.5	2.8
**Model composition**			
Chains	10	10	10
Non-hydrogen atoms	12037	11063	10138
Protein residues	767	762	722
Nucleotide	290	246	218
**Mean B-factors (Å^2)**			
Protein	51.82	34.28	60.97
Nucleotide	125.08	73.21	124.17
**R.m.s. deviations**			
Bond lengths (Å)	0.004	0.004	0.003
Bond angles (°)	0.620	0.614	0.600
**Validation**			
MolProbity score	1.59	1.64	1.75
Clash score	8.70	7.29	8.71
**Ramachandran plot**			
Outliers (%)	0	0	0
Allowed (%)	2.66	1.47	1.13
Favored (%)	97.34	98.53	98.87

Sample	**147-bp 5S rDNA nucleosome**	
Structures	147-bp 5S rDNA Nucleosome Closed	147-bp 5S rDNA Nucleosome Open I
Accession codes	PDB 9D3R; EMD-46545	PDB 9D3S; EMD-46546
Type of support	Quantifoil R2/1, UT, 300 Mesh, Copper (Cat No. Q350CR1-2nm)	
Microscope	Titan Krios G4 (Thermo Fisher Scientific)	
Detector	Falcon 4	
Energy filter	Selectris X	

Acceleration voltage (kV)	300	
Pixel size (Å)	0.935	
Total dose ($e^-/Å^2$)	50	
No. of micrographs recorded/used	5571/5291	
Symmetry	C1 (symmetry not imposed)	
No. of picked particles	3,022,989 (100%)	
No. of particles after 2D classifications	2,239,232 (74%)	
No. of particles after 3D classification with alignment	208,470 (7%)	
No. of particles in the final reconstruction after 3D classification without alignment	16,369 (0.5%)	80,111 (3%)
Global resolution (Å)	3.3	3.1
**Refinement**		
Model resolution (Å) (FSC threshold 0.143)	3.3	3.1
**Model composition**		
Chains	10	10
Non-hydrogen atoms	11858	10939
Protein residues	750	748
Nucleotide	290	246
**Mean B-factors ($Å^2$)**		
Protein	58.63	42.34
Nucleotide	142.43	97.53
**R.m.s. deviations**		
Bond lengths (Å)	0.008	0.011
Bond angles (°)	0.889	0.973
**Validation**		
MolProbity score	1.82	1.73
Clash score	12.72	14.35
**Ramachandran plot**		
Outliers (%)	0	0
Allowed (%)	3.27	2.32
Favored (%)	96.73	97.68

Sample	**Glutaraldehyde (GA)-crosslinked 167-bp 5S rDNA nucleosome**	**Formaldehyde (FA)-crosslinked 167-bp 5S rDNA nucleosome**	**Glutaraldehyde (GA)-crosslinked 147-bp 5S rDNA nucleosome**
Cryo-EM map and model availability	PDB 9D3N; EMD-46540	EMD-46541	PDB 9D3T; EMD-46547
Type of support	Quantifoil R2/1, UT, 300 Mesh, Copper (Cat No. Q350CR1-2nm)		
Microscope	Titan Krios G4 (Thermo Fisher Scientific)		
Detector	Falcon 4		
Energy filter	Selectris X		
Acceleration voltage (kV)	300		
Pixel size (Å)	0.935		
Total dose ($e^-/Å^2$)	50		
No. of micrographs recorded/used	5454/5152	6443/6280	5622/5352
Symmetry	C1 (symmetry not imposed)		

No. of picked particles	1,939,825 (100%)	3,344,880 (100%)	2,164,687 (100%)
No. of particles after 2D classifications	479,048 (25%)	1,493,665 (45%)	1,076,644 (50%)
No. of particles after 3D classification with alignment	85,946 (4%)	308,514 (9%)	184,144 (9%)
No. of particles in the final reconstruction after 3D classification without alignment	35,903 (2%)	224,003 (7%)	53,805 (2%)
Global resolution (Å)	3.0	2.6 (overestimated value due to preferential views)	2.8
**Refinement**			
Model resolution (Å) (FSC threshold 0.143)	3.0	N/A	2.8
**Model composition**			
Chains	10		10
Non-hydrogen atoms	9401		9820
Protein residues	697		716
Nucleotide	192		206
**Mean B-factors (Å^2)**			
Protein	46		40.44
Nucleotide	126.29		97.83
**R.m.s. deviations**			
Bond lengths (Å)	0.003		0.005
Bond angles (°)	0.652		0.729
**Validation**			
MolProbity score	1.58		2.19
Clash score	10.38		10.36
**Ramachandran plot**			
Outliers (%)	0		0
Allowed (%)	220		3.00
Favored (%)	97.80		97.00

Supplemental Table S1. Cryo-EM data collection, refinement, and validation statistics.

		Sample				
		167-bp 5S rDNA Nucleosome	147-bp 5S rDNA Nucleosome	Glutaraldehyde (GA)-crosslinked 167-bp 5S rDNA Nucleosome	Formaldehyde (FA)-crosslinked 167-bp 5S rDNA Nucleosome	Glutaraldehyde (GA)-crosslinked 147-bp 5S rDNA Nucleosome
**5S rDNA Nucleosome Structural State Based on DNA Flexibility**	Closed (Without Unwrapped Ends)	5%	36%	0%	0%	0%
	One End Unwrapped	95%<br>60% (Open I-like) 35% (Open II-like)	44% (Open I-like)	0%	0%	12%
	Both Ends Unwrapped	0%	0%	100%	100%	73%
	Heterogeneous Classes with Challenging Interpretation	0%	20%	0%	0%	15%
	3D Classification Without Alignment Input Particles	100%	100%	100%	100%	100%

Supplemental Table S2. Percentage distribution of particles across 5S rDNA nucleosome structural states according to DNA flexibility. Particles selected after 2D classification and 3D classification with alignment were used in 3D classification without alignment and correspond to 100%.

Supplemental Movie S1. 3D variability analysis of the crosslinked Dsup-bound nucleosome after map refinement using an initial reference without one DNA end.

Supplemental Movie S2. 3D variability analysis of the crosslinked Dsup-bound nucleosome after map refinement using an initial reference without both DNA ends.

Supplemental Movie S3. 167-bp 5s rDNA nucleosome dynamics: downstream DNA opening.

Supplemental Movie S4. 167-bp 5s rDNA nucleosome dynamics: upstream DNA opening.

Supplemental Materials and Methods

Purification of recombinant human histones and reconstitution of histone octamers

The coding sequences of the four human core histones were each individually cloned into the pET11a vector (Novagen). Each histone was synthesized in *Escherichia coli* BL21(DE3) cells (Novagen) and purified from inclusion bodies by the method of Luger et al. (1999) with some modifications as follows. After induction with IPTG at a final concentration of 0.4 mM at 37 °C for 2 h, cells (1 g) were resuspended in 25 mL of cold buffer W (50 mM Tris-HCl, pH 7.5, 0.1 M NaCl, 1 mM EDTA, 100 µg/mL lysozyme, 1 mM benzamidine, 5 mM 2-mercaptoethanol, and 0.25 mM PMSF) and subjected to two freeze-thaw cycles. Next, the cell suspension was centrifuged (14,000 rpm for 15 min at 4 °C; Fiberlite F21S-8x50y rotor), and the resulting pellet was resuspended in 25 mL of cold TW buffer [50 mM Tris-HCl, pH 7.5, 0.1 M NaCl, 1 mM EDTA, 1% (v/v) Triton X-100, 1 mM benzamidine, 5 mM 2-mercaptoethanol, and 0.25 mM PMSF] and dispersed by using a glass Wheaton Dounce homogenizer with a loose B pestle. Then, the cells were lysed on ice by 10 sonication cycles (each for 15 s ON and 15 s OFF, 20% output; Branson Sonifier 450). The pellet containing the inclusion bodies was recovered by centrifugation (14,000 rpm for 15 min at 4 °C; Fiberlite F21S-8x50y rotor) and washed an additional two times with cold TW buffer followed by three washes with cold buffer W. The pellet was minced in 0.3 mL of DMSO, incubated for 30 min at 22 °C, suspended in 5 mL of buffer G (20 mM Tris-HCl, pH 7.5, 7 M guanidine-HCl, and 10 mM DTT), and incubated at 22 °C for 1 h on a nutator. The soluble fraction containing the histone was recovered by centrifugation (14,000 rpm for 15 min at 20 °C; Fiberlite F21S-8x50y rotor) and dialyzed (molecular weight cutoff: 3.5 kDa) against three changes (each at 4 °C for 2 h) of buffer U (10 mM Tris-HCl, pH 7.5, 1 mM EDTA, 7 M urea, 5 mM 2-mercaptoethanol, and 0.25 mM PMSF) containing 0.2 M NaCl. After dialysis, the insoluble material was removed by centrifugation (18,000 rpm for 10

min at 10 °C; Fiberlite F21S-8x50y rotor), and the histones were purified from the supernatant by tandem Q Sepharose High Performance followed by SP Sepharose High Performance ion-exchange chromatography. Briefly, after sample loading, the Q Sepharose High Performance column (5 mL packed resin) was removed, and the SP Sepharose High Performance column (5 mL packed resin) was washed with 15 mL of buffer U containing 0.2 M NaCl followed by 5 mL of buffer U containing 0.25 M NaCl. Then, the histones were eluted with a linear gradient of 0.25 M to 0.5 M NaCl in buffer U over 50 mL total volume. The peak fractions containing the most highly purified histones were pooled and dialyzed (molecular weight cutoff: 3.5 kDa) against three changes of 2 L of cold water containing 5 mM 2-mercaptoethanol and 0.2 mM PMSF (the first and the last dialysis steps for 2 h, and the second step overnight at 4 °C). After dialysis, the insoluble materials were removed by centrifugation, and the histones were lyophilized to dryness (medium drying rate; Savant Speed Vac Plus) and stored at –80 °C. The molecular mass of each purified histone protein was determined by mass spectrometry (Molecular Mass Spectrometry Facility, UCSD). This analysis confirmed the integrity of the histones and showed that all four histones lacked the N-terminal initiating methionine residue. The numbering of the histone residues throughout the figures and manuscript begins at the first residue following the initiating methionine.

The sequences of the human core histone proteins used in this study are as follows. Histone H2A2A (NCBI Reference Sequence NP_003500.1; UniProt Q6FI13): MSGRGKQGGKARAKAKSRSSRAGLQFPVGRVHRLLRKGNYAERVGAGAPVYMAAVLEYLTAEILELAGNAARDNKKTRIIPRHLQLAIRNDEELNKLLGKVTIAQGGVLPNIQAVLLPKKTESHHKAKGK; histone H2B1C (NCBI Reference Sequence NP_001368918.1; UniProt P62807): MPEPAKSAPAPKKGSKKAVTKAQKKDGKKRKRSRKESYSVYVYKVLKQVHPDTGISSKAMGIMNSFVNDIFERIAGEASRLAHYNKRSTITSREIQTAVRLLLPGELAKHAVSEGTKAVTKYTSSK; histone H3.2 (NCBI Reference Sequence NP_001005464.1; UniProt Q71DI3):

MARTKQTARKSTGGKAPRKQLATKAARKSAPATGGVKKPHRYRPGTVALREIRRYQKSTELLIRKLPFQRLVREIAQDFKTDLRFQSSAVMALQEASEAYLVGLFEDTNLCAIHAKRVTIMPKDIQLARRIRGERA; histone H4 (NCBI Reference Sequence NP_001029249.1; UniProt P62805): MSGRGKGGKGLGKGGAKRHRKVLRDNIQGITKPAIRRLARRGGVKRISGLIYEETRGVLKVFLENVIRDAVTYTEHAKRKTVTAMDVVYALKRQGRTLYGFGG.

To reconstitute histone octamers, each lyophilized histone was dissolved in buffer G at 22 °C for 1 h. The absorbance at 280 nm of each histone sample was measured with a spectrophotometer (Nanodrop One; Thermo Scientific), and the histone concentration was determined by using the following molar extinction coefficients at 280 nm and molecular masses: H2A2A, 4470 M^{-1} cm^{-1}, 14,095 g/mol; H2B1C, 7450 M^{-1} cm^{-1}, 13,906 g/mol; H3.2, 4470 M^{-1} cm^{-1}, 15,388 g/mol; H4, 5960 M^{-1} cm^{-1}, 11,367 g/mol. Then, a histone mix was prepared by combining an equimolar ratio of H3 and H4 with a 25% molar excess of both H2A and H2B (*i.e.*, molar ratio 1.0:1.0:1.25:1.25 of H3:H4:H2A:H2B). The final protein concentration was adjusted to 1 mg/mL with buffer G, and the mixture (3 mL maximum volume) was dialyzed (molecular weight cutoff: 3.5 kDa) against three changes of 1 L of buffer R (10 mM Tris-HCl, pH 7.5, 2 M NaCl, 1 mM EDTA, and 5 mM 2-mercaptoethanol) at 4 °C (the first and the last dialysis steps for 2 h each, and the second step overnight). The insoluble material was removed by centrifugation at 13,200 rpm for 10 min at 4 °C (Eppendorf 5415R), and the supernatant was concentrated by using a protein concentrator (molecular weight cutoff: 50 kDa). The concentrated sample was centrifuged at 13,200 rpm for 10 min at 4 °C (Eppendorf 5415R), and the supernatant (about 0.24 mL) was loaded onto a Superose 12 HR 10/30 size exclusion column (24 mL packed resin; GE Healthcare). The histone octamers were eluted with 24 mL of buffer R (fraction size: 0.25 mL). The peak fractions containing equimolar amounts of each histone were pooled and then dialyzed against three changes of storage buffer [10 mM Hepes-K^+, pH 7.6, 1 mM EDTA, 10 mM KCl, 10% (v/v) glycerol, and 1 mM DTT] at 4 °C (the first and the last dialysis steps for 2 h

each, and the second step overnight). The histones were frozen in liquid nitrogen and stored at –80 °C.

Purification of recombinant Dsup proteins and recombinant HMGN proteins

Recombinant His6- and FLAG-tagged versions of the full-length *R. varieornatus* Dsup protein, the C-terminus regions of R. *varieornatus* Dsup (residues 306-445), *H. exemplaris* Dsup (residues 183-328), and *P. richtersi* Dsup-like (GenBank GFGY01013230.1; residues 182-366) proteins were purified by the nondenaturing method described in Chavez et al. (2019).

The coding sequence of human HMGN2 protein was codon optimized for expression in *E. coli* and cloned into vector pET11a (Novagen). BL21(DE3) cells were transformed with the recombinant DNA construct, and protein synthesis was induced by the addition of IPTG to a final concentration of 0.4 mM at 30 °C for 1.5 h. Then, HMGN2 was purified by cation-exchange chromatography as described in Paranjape et al. (1995) with some modifications. Briefly, 1 g of bacterial cells was resuspended in 6 mL of cold buffer L (50 mM Hepes-K^+, pH 7.6, 0.1 M NaCl, 1 mM benzamidine, 1 mM DTT, 4 µg/mL leupeptin, 4 µg/mL aprotinin, 1 µg/mL pepstatin, and 0.2 mM PMSF) and then lysed by sonication on ice (10 cycles 15 s ON/ 15 s OFF, 20% output; Branson Sonifier 450). The insoluble fraction was recovered by centrifugation (13,000 rpm for 10 min at 4 °C; Fiberlite F21S-8x50y rotor). The pellet containing HMGN2 was resuspended in 4 mL of cold buffer L, and the protein was extracted by the addition of 12 M HCl to a final concentration of 0.77 M. After incubation at 4 °C for 1 h on a rotating wheel, the insoluble material was removed by centrifugation (13,000 rpm for 10 min at 4 °C; Fiberlite F21S-8x50y rotor), and the supernatant containing HMGN2 was dialyzed (molecular weight cutoff: 3.5 kDa) against two changes of 2 L of buffer H (50 mM Hepes-K^+, pH 7.6, 0.1 M NaCl, 1 mM benzamidine, 1 mM DTT, and 0.2 mM PMSF), with each dialysis step at 4 °C for 2 h. After dialysis, the insoluble material was removed by centrifugation (18,000 rpm for 10 min at 4 °C;

Fiberlite F21S-8x50y rotor), and the supernatant containing HMGN2 was loaded onto a CM Sepharose Fast Flow column (20 mL packed resin; column: XK16/20 GE Healthcare). The column was washed with 80 mL of buffer H, and then the protein was eluted with a 0.15 M to 0.35 M NaCl gradient in buffer H over 60 mL followed by a 0.35 M to 0.55 M NaCl gradient in buffer H over 100 mL. The peak fractions containing highly pure HMGN2 were pooled and dialyzed against two changes of buffer S [10 mM Hepes-K^+, pH 7.6, 10 mM KCl, 0.5 mM EGTA, 1.5 mM $MgCl_2$, 10% (v/v) glycerol, 1 mM DTT, 10 mM glycerol 2-phosphate, 1 mM benzamidine, and 0.2 mM PMSF] at 4 °C (the first dialysis step for 2 h and the second dialysis step overnight). The protein was frozen in liquid nitrogen and stored at –80 °C. The molecular mass of the human HMGN2 protein was determined by mass spectrometry (Molecular Mass Spectrometry Facility, UCSD). This analysis confirmed the integrity of the protein and showed that HMGN2 lacks the N-terminal initiating methionine residue. The numbering of the HMGN2 residues throughout the figures and manuscript begins at the first residue following the initiating methionine. The human HMGN2 protein sequence is as follows (NCBI Reference Sequence NP_005508.1; UniProt P05204):
MPKRKAEGDAKGDKAKVKDEPQRRSARLSAKPAPPKPEPKPKKAPAKKGEKVPKGKKGKADA
GKEGNNPAENGDAKTDQAQKAEGAGDAK.

The coding sequence of the human HMGN5 protein containing His6 (N-terminal) and FLAG (C-terminal) tags was codon optimized for expression in *E. coli* and cloned into the pET21b vector (Novagen). The His6-FLAG tagged HMGN5 protein was synthesized in Rosetta(DE3)pLysS cells (Novagen) and purified by Ni-NTA affinity chromatography as described for Dsup in Chavez et al. (2019) with the following modifications. First, the lysis buffer was substituted with buffer HL [50 mM Hepes-Na^+, pH 7.8, 0.1 mM EDTA, 0.1 M NaCl, 0.01% (v/v) NP-40, 5% (v/v) glycerol, 10 mM 2-mercaptoethanol, 1 µg/mL pepstatin, 1 µg/mL leupeptin, 1 mM benzamidine, 0.5 mM PMSF, 300 µg/mL lysozyme, and 100 µg/mL Pefabloc (4-(2-aminoethyl)-benzene-1-sulfonyl fluoride)]. Second, after sonication in buffer HL, the

protein was extracted from the soluble material by the addition of 12 M HCl to a final concentration of 0.75 M for 30 min on ice. Then, the supernatant containing the protein was recovered by centrifugation (18,000 rpm for 15 min at 4 °C; Fiberlite F21S-8x50y rotor). The solution was neutralized by the addition of 5 M NaOH, and 1 M Tris-HCl, pH 8.0, and 1 M imidazole were added to 50 mM and 20 mM final concentrations, respectively, before loading onto a Ni-NTA column. The human HMGN5 protein sequence is as follows (NCBI Reference Sequence NP_110390.1; UniProt P82970):

MPKRKAAGQGDMRQEPKRRSARLSAMLVPVTPEVKPKRTSSSRKMKTKSDMMEENIDTSAQ
AVAETKQEAVVEEDYNENAKNGEAKITEAPASEKEIVEVKEENIEDATEKGGEKKEAVAAEVKN
EEEDQKEDEEDQNEEKGEAGKEDKDEKGEEDGKEDKNGNEKGEDAKEKEDGKKGEDGKGN
GEDGKEKGEDEKEEEDRKETGDGKENEDGKEKGDKKEGKDVKVKEDEKEREDGKEDEGGN
EEEAGKEKEDLKEEEEGKEEDEIKEDDGKKEEPQSIV.

Reconstitution of nucleosomes as well as Dsup-nucleosome and HMGN-nucleosome complexes

Nucleosomes (mononucleosomes) containing recombinant human histones octamers and 147 bp or 167 bp of *Xenopus borealis* 5S rDNA, or 147 bp of the 601 DNA sequence were reconstituted by step-wise salt dialysis as described in Chavez et al. (2019) with the following modifications. Briefly, the DNA was combined with the histone octamers in buffer M [25 mM Hepes-K^+, pH 7.6, 0.1 mM EDTA, 0.01% (v/v) NP-40, and 1 M NaCl]. The histone-DNA mixture was incubated for 30 min on ice and then dialyzed (molecular weight cutoff: 3.5 kDa) against three changes (each at 22 °C for 2.5 h) of buffer HE (25 mM Hepes-K^+, pH 7.6, and 0.1 mM EDTA) containing decreasing concentrations of NaCl (0.8 M NaCl, 0.6 M NaCl, and 50 mM NaCl). Then, the samples were heated at 58 °C for 10 min to facilitate the most stable nucleosome position, and the quality of the nucleosomes was assessed by native

(nondenaturing) 4.5% polyacrylamide gel electrophoresis. The reconstituted nucleosomes were stored at 4 °C prior to sample preparation for cryo-EM studies. The 147-bp 601-DNA sequence is as follows:

CTGGAGAATCCCGGTGCCGAGGCCGCTCAATTGGTCGTAGACAGCTCTAGCACCGCTTAAACGCACGTACGCGCTGTCCCCCGCGTTTTAACCGCCAAGGGGATTACTCCCTAGTCTCCAGGCACGTGTCAGATATATACATCCTGT. The 147-bp *Xenopus borealis* somatic 5S rDNA gene fragment (Peterson et al. 1980; Rhodes et al. 1985) is the central 147-bp nucleosome positioning sequence that was precisely mapped in Fei et al. (2018). The sequence of the 167-bp *Xenopus borealis* 5S rDNA fragment (with the central 147-bp positioning sequence underlined) is as follows:

TCAAGGCCGGGCTTGTTTTCCTGCCTGGGGGAAAAGACCCTGGCATGGGGAGGAGCTGGGCCCCCCCCAGAAGGCAGCACAAGGGGAGGAAAAGTCAGCCTTGTGCTCGCCTACGGCCATACCACCCTGAAAGTGCCCGATATCGTCTGATCTCGGAAGCCAAGCAG.

To reconstitute the protein-nucleosome complexes (2.1 μM final concentration), the purified recombinant Dsup, HMGN2, or HMGN5 proteins were combined with nucleosomes in binding buffer [25 mM Hepes-K^+, pH 7.6, 0.1 mM EDTA, 90 mM KCl, 1.8% (v/v) glycerol, and 0.01% (v/v) NP-40] and incubated at 27 °C for 1 h. The molar ratios of nucleosome:Dsup, nucleosome:HMGN2, and nucleosome:HMGN5 in the binding reactions were 1:3, 1:3, and 1:2, respectively. In addition, control samples of nucleosomes (2.1 μM final concentration) that did not contain Dsup or the HMGN proteins were processed in parallel. The formation of the protein-nucleosome complexes was assessed by analyzing a small portion (1 pmol) of the samples by 4.5% polyacrylamide native gel electrophoresis.

Grid preparation for cryo-EM analysis

Holey grids with continuous carbon on top (Quantifoil® R 2/1, UT, 300 Mesh, Cu or Quantifoil® R 2/1, UT, 200 Mesh, Cu (Electron Microscopy Sciences) were glow-discharged for 30 s at 20-30 mA. 2.6 µL to 3 µL of sample was applied to the grids and flash-frozen using a Vitrobot Mark IV (FEI; 95% humidity, 4 °C, blot force 4, blot time 4). Non-crosslinked samples were used at concentrations of 0.5-0.6 µM, whereas HMGN2 or Dsup crosslinked to nucleosomes were at 1.8 µM or 0.9 µM, respectively. All samples were kept on ice prior to vitrification.

Cryo-EM data collection

Data were collected on the in-house Titan Krios G4 (Thermo Fisher Scientific) operated at 300 kV and equipped with a Falcon 4 direct electron detector (Thermo Fisher Scientific) and a Selectris X energy filter (Thermo Fisher Scientific). A nominal magnification of 130,000× (object pixel size of 0.935 Å), a total dose of 50 $e^{-}/Å^{2}$ and an applied defocuses range of –0.6 to –2.2 µm were used.

Cryo-EM image processing

In CryoSPARC Live, on-the-fly Patch Motion Correction and Patch CTF Estimation jobs were used to apply the gain reference, align and estimate the CTF of the collected movie frames (Punjani et al. 2017). Micrographs with a CTF fit worse than 3 Å were always discarded. Depending on the dataset, the 'Relative ice thickness' (greater than 1.07) and 'Total motion' (greater than 20 pixels) parameters were also used to remove micrographs, as well as manual inspection. In CryoSPARC (Punjani et al. 2017), template picking followed of rounds of 2D classifications with 4x binned particles allowed the selection of 'good' particles, which were used for training Topaz (Bepler et al. 2019). Several rounds of 2D classification and particle curation culminated in the selection of a particle stack. For free and HMGN2-bound 5S nucleosomes,

this particle stack was re-extracted at 1.87 Å/pix (2x binned). pyem/csparc2star.py (https://doi.org/10.5281/zenodo.3576630) was used to convert the CryoSPARC readable particle file to RELION's readable format (.star) and 3D classifications with alignment were performed in RELION-3.1 or RELION-4.0 (Scheres 2012; Scheres 2016). A soft masked, 60 Å low-pass filtered initial reference was used, as well as a 113 to 120 Å circular mask around each particle. Initially, 25 iterations with exhaustive angular searches (angular sampling interval 7.5°, offset search range 5 pix, offset search step 1 pix) and regularization parameter T = 4 were run; then the 3D classification was continued 10 to 25 extra iterations with narrower angular searches (angular sampling interval 3.7°, offset search range 3 to 4 pix, offset search step 0.8 to 1 pix). The output class with best nucleosome features was 3D refined in RELION (Scheres, 2012) or CryoSPARC (Punjani et al. 2020), and aligned particles were 3D classified without alignment in RELION-3.1 or RELION-4.0. The initial reference was low-pass filtered at 10 to 15 Å, and it was softly masked; 2 to 10 classes were used. In the case of the HMGN2-bound 167-bp 5S rDNA nucleosome, 3D classification without alignment was not performed. Instead, a second round of 3D classification with alignment was run without masking the initial reference. After 3D classifications, particles from selected classes were imported to CryoSPARC and subjected to Non-uniform refinement in CryoSPARC (Punjani et al. 2020) (per-micrograph CTF refinement was always used; sometimes, enabling 'Enforce non-negativity' slightly improved the map). Automatically sharpened maps from CryoSPARC's Non-uniform refinement were used in ChimeraX (Goddard et al. 2018) to make figures.

Image processing of 601-DNA nucleosomes bound to Dsup was performed in CryoSPARC. A 2D classification-curated particle stack was extracted at the original pixel size (0.935 Å) and refined using an initial 3D reference with one DNA end wrapped around the octamer (Supplemental Fig. S1 and Supplemental Movie S1). It should be noted that starting the 3D refinement with a 3D reference lacking both DNA ends poorly handles 2-fold averaging around the dyad axis, misrepresenting nucleosome breathing in crosslinked Dsup-bound

nucleosomes during subsequent 3D variability analysis (Supplemental Movie S2). After refinement, 3D classification into 6 classes, without alignment, yielded one class whose particles were refined to reconstruct Dsup-bound nucleosome structure I. Particles from 4 other classes were joined and refined to reconstruct Dsup-bound nucleosome structure II.

3D variability analysis

After 3D classification with alignment in RELION, selected particles of the native 167-bp 5S rDNA nucleosome were imported to CryoSPARC and a non-uniform refinement run. 3D variability analysis (Punjani and Fleet 2021) was performed with the particles oriented as in the previous refinement, applying a 3D soft mask around the nucleosome and solving three orthogonal principal modes. Results were displayed using 'Output mode - intermediates', downsampling box size from 208 to 104 pixels and low-pass filtering the maps at 5 Å. All remaining parameters were left with the default values. Frames correspondent to the first (main) and second component of variability were used to record movies in ChimeraX (Goddard et al. 2018) (Supplemental Movies S1 and S2).

To visualize the effect of the initial reference in 3D refinements (density absent for one DNA end versus density absent for the two DNA ends) on 2-fold averaging of Dsup-bound nucleosomes, 3D variability analysis was performed as described and movies recorded using the main component of variability (Supplemental Movies S1 and S2).

Cryo-EM: determination of the nucleosomal DNA orientation

For pdb 3LZ0, 25-bp centered on the dyad (DNA position 0) were mutated to the corresponding sequence of the 5S rDNA using ChimeraX (Goddard et al. 2018). For each cryo-EM map whose DNA orientation we analyzed, this coordinates file was rigid-fitted into the map in the two possible orientations related through a 180° rotation over the dyad axis. Separately, each orientation was real-space refined in Coot 0.9.4 (Emsley et al. 2010) followed of Phenix 1.20

(Afonine et al. 2018). Refined DNAs were opened in Chimera 1.15 (Pettersen et al. 2004) to obtain a list of atomic B-factors (Urzhumtsev et al. 2022), excluding the P, OP1, OP2, O5’ and O3’ atoms. These phosphate backbone atoms were excluded because they are subject to greater variations in the value of the B-factors (for instance, the phosphate backbone facing the octamer has in principle much lower B-factors than the atoms facing the solvent). However, in our experience, excluding the P, OP1, OP2, O5’ and O3’ atoms should not significantly alter the final B-factor analysis. Using Microsoft Excel (https://office.microsoft.com/excel), atomic B-factors were averaged per-bp and plotted versus the bp number. Note that to compare the two DNA orientations one was flipped horizontally in the graphic (this meaning that bp −12 to +12 of a given orientation need to overlap with bp +12 to −12 of the opposite orientation).

Cryo-EM: atomic modelling

In Chimera 1.15 (Pettersen et al. 2004), the nucleotides and amino acids of pdb 7LYA were mutated to match our DNA and histone sequences. Chains were renumbered. The resulting coordinates file was rigid-fitted in the DNA orientation previously found. If needed, DNA segments without EM density in the maps filtered according to local resolution estimation were deleted. Each nucleotide and amino acid was fitted into the density using Coot 0.9.4 (Emsley et al. 2010). When applicable, some amino acids or side chains were removed or de novo built. Real space refinement in Phenix 1.20 (Afonine et al. 2018) was run. 2 to 3 cycles of Coot 0.9.4 and Phenix 1.20 were performed.

Supplemental References

Abramson J, Adler J, Dunger J, Evans R, Green T, Pritzel A, Ronneberger O, Willmore L, Ballard AJ, Bambrick J, et al. 2024. Accurate structure prediction of biomolecular interactions with AlphaFold 3. *Nature* **630**: 493–500.

Afonine PV, Poon BK, Read RJ, Sobolev OV, Terwilliger TC, Urzhumtsev A, Adams PD. 2018. Real-space refinement in PHENIX for cryo-EM and crystallography. *Acta Crystallogr D Struct Biol* **74**: 531–544.

Altschul SF, Madden TL, Schäffer AA, Zhang J, Zhang Z, Miller W, Lipman DJ. 1997. Gapped BLAST and PSI-BLAST: a new generation of protein database search programs. *Nucleic Acids Res* **25**: 3389–3402.

Bepler T, Morin A, Rapp M, Brasch J, Shapiro L, Noble AJ, Berger B. 2019. Positive-unlabeled convolutional neural networks for particle picking in cryo-electron micrographs. *Nat Methods* **16**: 1153–1160.

Chavez C, Cruz-Becerra G, Fei J, Kassavetis GA, Kadonaga JT. 2019. The tardigrade damage suppressor protein binds to nucleosomes and protects DNA from hydroxyl radicals. *eLife* **8**: e47682.

Emsley P, Lohkamp B, Scott WG, Cowtan K. 2010. Features and development of Coot. *Acta Crystallogr D Biol Crystallogr* **66**: 486–501.

Fei J, Ishii H, Hoeksema MA, Meitinger F, Kassavetis GA, Glass CK, Ren B, Kadonaga JT. 2018. NDF, a nucleosome-destabilizing factor that facilitates transcription through nucleosomes. *Genes Dev* **32**: 682–694.

Goddard TD, Huang CC, Meng EC, Pettersen EF, Couch GS, Morris JH, Ferrin TE. 2018. UCSF ChimeraX: Meeting modern challenges in visualization and analysis. *Protein Sci* **27**: 14–25.

Hashimoto T, Horikawa DD, Saito Y, Kuwahara H, Kozuka-Hata H, Shin-I T, Minakuchi Y, Ohishi K, Motoyama A, Aizu T, et al. 2016. Extremotolerant tardigrade genome and improved radiotolerance of human cultured cells by tardigrade-unique protein. *Nat Commun* **7**: 12808.

Luger K, Rechsteiner TJ, Richmond TJ. 1999. Preparation of nucleosome core particle from recombinant histones. *Methods Enzymol* **304**: 3–19.

McGinty RK, Tan S. 2021. Principles of nucleosome recognition by chromatin factors and enzymes. *Curr Opin Struct Biol* **71**: 16–26.

Paranjape SM, Krumm A, Kadonaga JT. 1995. The HMG17 is a chromatin-specific transcriptional coactivator that increases the efficiency of transcription initiation. *Genes Dev* **9**: 1978–1991.

Peterson RC, Doering JL, Brown DD. 1980. Characterization of two xenopus somatic 5S DNAs and one minor oocyte-specific 5S DNA. *Cell* **20**: 131–141.

Pettersen EF, Goddard TD, Huang CC, Couch GS, Greenblatt DM, Meng EC, Ferrin TE. 2004. UCSF Chimera – a visualization system for exploratory research and analysis. *J Comput Chem* **25**: 1605–1612.

Pieler T, Hamm J, Roeder RG. 1987. The 5S gene internal control region is composed of three distinct sequence elements, organized as two functional domains with variable spacing. *Cell* **48**: 91–100.

Punjani A, Fleet DJ. 2021. 3D variability analysis: Resolving continuous flexibility and discrete heterogeneity from single particle cryo-EM. *J Struct Biol* **213**: 107702.

Punjani A, Rubinstein JL, Fleet DJ, Brubaker MA. 2017. cryoSPARC: algorithms for rapid unsupervised cryo-EM structure determination. *Nat Methods* **14**: 290–296.

Punjani A, Zhang H, Fleet DJ. 2020. Non-uniform refinement: adaptive regularization improves single-particle cryo-EM reconstruction. *Nat Methods* **17**: 1214–1221.

Rhodes D. 1985. Structural analysis of a triple complex between the histone octamer, a *Xenopus* gene for 5S RNA and transcription factor IIIA. *EMBO J* **4**: 3473–3482.

Robert X, Gouet P. 2014. Deciphering key features in protein structures with the new ENDscript server. *Nucleic Acids Res* **42**: W320–324.

Scheres SH. 2012. RELION: implementation of a Bayesian approach to cryo-EM structure determination. *J Struct Biol* **180**: 519–530.

Scheres SH. 2016. Processing of Structurally Heterogeneous Cryo-EM Data in RELION. *Methods Enzymol* **579**: 125–157.

Sievers F, Wilm A, Dineen D, Gibson TJ, Karplus K, Li W, Lopez R, McWilliam H, Remmert M, Söding J, Thompson JD, Higgins DG. 2011. Fast, scalable generation of high-quality protein multiple sequence alignments using Clustal Omega. *Mol Syst Biol* **7**: 539.

Urzhumtsev AG, Urzhumtseva LM, Lunin VY. 2022. Direct calculation of cryo-EM and crystallographic model maps for real-space refinement. *Acta Crystallogr D Struct Biol* **78**: 1451–1468.

Wu TD, Brutlag DL. 1996. Discovering empirically conserved amino acid substitution groups in databases of protein families. *Proc Int Conf Intell Syst Mol Biol* **4**: 230–240.
