# Supplementary material for: Structural basis of nucleosome recognition by the conserved Dsup and HMGN nucleosome-binding motif
Source: Genes Dev. 2025 Oct 1;39(19-20):1155–61. doi: 10.1101/gad.352720.125 (PMC12487698; doi:10.1101/gad.352720.125)
Supplement: Supplement 6 [file Supplemental_Table_S1_Movies_S1_S4_.docx]

| Sample | **147-bp 601 nucleosome crosslinked to Dsup** | | | **167-bp 5S rDNA nucleosome crosslinked to HMGN2** | | | **167-bp 5S rDNA nucleosome crosslinked to HMGN5** |
| --- | --- | --- | --- | --- | --- | --- | --- |
| Structures | Dsup-bound Nucleosome Structure I | Dsup-bound Nucleosome Structure II | |  | | |  |
| Accession codes | PDB 9D3L  EMD-46537 | PDB 9D3K  EMD-46536 | | PDB 9D3M  EMD-46538 | | | EMD-46539 |
| Type of support | Quantifoil R2/1, UT, 300 Mesh, Copper (Cat No. Q350CR1-2nm) | | | | | | |
| Microscope | Titan Krios G4 (Thermo Fisher Scientific) | | | | | | |
| Detector | Falcon 4 | | | | | | |
| Energy filter | Selectris X | | | | | | |
| Acceleration voltage (kV) | 300 | | | | | | |
| Pixel size (Å) | 0.935 | | | | | | |
| Total dose  (e^-^/Å^2^) | 50 | | | | | | |
| No. of micrographs recorded/used | 10949/10056 | | | 13833/11761 | | | 13648/12318 |
| Symmetry | C1 (symmetry not imposed) | | | | | | |
| No. of picked particles | 3,767,974 (100%) | | | 4,738,442 (100%) | | | 2,166,837 (100%) |
| No. of particles after 2D classification | 644,478 (17%) | | | 1,079,681 (50%) | | | 2,279,409 (48%) |
| No. of particles after 3D classification with alignment | N/A | | | 55,942 (3%) | | | 220,875 (5%) |
| No. of particles in the final reconstruction | 194,251 (5%) | 371,055 (10%) | | 55,942 (3%) | | | 220,875 (5%) |
| Global resolution (Å) | 2.8 | 2.7 | | 2.9 | | | 2.9 |
|  | | | | | | | |
| **Refinement** |  | | | | | | |
| Model resolution (Å)  (FSC threshold 0.143) | 2.8 | 2.7 | | 2.9 | | | N/A |
| **Model composition** |  |  | |  | | |  |
| Chains | 12 | 12 | | 12 | | |  |
| Non-hydrogen atoms | 10994 | 9677 | | 10203 | | |  |
| Protein residues | 752 | 742 | | 765 | | |  |
| Nucleotide | 248 | 188 | | 202 | | |  |
| **Mean B-factors (Å^2^)** |  |  | |  | | |  |
| Protein | 48.22 | 30.35 | | 63.46 | | |  |
| Nucleotide | 99.18 | 61.59 | | 130.20 | | |  |
| **R.m.s. deviations** |  |  | |  | | |  |
| Bond lengths (Å) | 0.004 | 0.004 | | 0.005 | | |  |
| Bond angles (°) | 0.659 | 0.654 | | 0.539 | | |  |
| **Validation** |  |  | |  | | |  |
| MolProbity score | 1.77 | 1.84 | | 1.60 | | |  |
| Clash score | 7.52 | 8.03 | | 6.53 | | |  |
| **Ramachandran plot** |  |  | |  | | |  |
| Outliers (%) | 0.00 | 0.14 | | 0.00 | | |  |
| Allowed (%) | 1.50 | 2.49 | | 2.28 | | |  |
| Favored (%) | 98.50 | 97.37 | | 97.72 | | |  |
|  | | | | | | | |
| Sample | **167-bp 5S rDNA nucleosome** | | | | | | |
| Structures | 167-bp 5S rDNA Nucleosome Closed | | 167-bp 5S rDNA Nucleosome Open I | | | 167-bp 5S rDNA Nucleosome Open II | |
| Accession codes | EMD - 46542  PDB 9D3O | | PDB 9D3P  EMD-46543 | | | PDB 9D3Q  EMD-46544 | |
| Type of support | Quantifoil R2/1, UT, 300 Mesh, Copper (Cat No. Q350CR1-2nm) | | | | | | |
| Microscope | Titan Krios G4 (Thermo Fisher Scientific) | | | | | | |
| Detector | Falcon 4 | | | | | | |
| Energy filter | Selectris X | | | | | | |
| Acceleration voltage (kV) | 300 | | | | | | |
| Pixel size (Å) | 0.935 | | | | | | |
| Total dose  (e^-^/Å^2^) | 50 | | | | | | |
| No. of micrographs recorded/used | 5005/4708 | | | | | | |
| Symmetry | C1 (symmetry not imposed) | | | | | | |
| No. of picked particles | 2,263,256 (100%) | | | | | | |
| No. of particles after 2D classifications | 1,549,385 (68%) | | | | | | |
| No. of particles after 3D classification with alignment | 280,478 (12%) | | | | | | |
| No. of particles in the final reconstruction after 3D classification without alignment | 12,789 (0.6%) | | 280,478 (12%) | | | 24,628 (1%) | |
| Global resolution (Å) | 3.0 | | 2.5 | | | 2.8 | |
|  | | | | | | | |
| **Refinement** |  | |  | | |  | |
| Model resolution (Å)  (FSC threshold 0.143) | 3.0 | | 2.5 | | | 2.8 | |
| **Model composition** |  | |  | | |  | |
| Chains | 10 | | 10 | | | 10 | |
| Non-hydrogen atoms | 12037 | | 11063 | | | 10138 | |
| Protein residues | 767 | | 762 | | | 722 | |
| Nucleotide | 290 | | 246 | | | 218 | |
| **Mean B-factors (Å^2^)** |  | |  | | |  | |
| Protein | 51.82 | | 34.28 | | | 60.97 | |
| Nucleotide | 125.08 | | 73.21 | | | 124.17 | |
| **R.m.s. deviations** |  | |  | | |  | |
| Bond lengths (Å) | 0.004 | | 0.004 | | | 0.003 | |
| Bond angles (°) | 0.620 | | 0.614 | | | 0.600 | |
| **Validation** |  | |  | | |  | |
| MolProbity score | 1.59 | | 1.64 | | | 1.75 | |
| Clash score | 8.70 | | 7.29 | | | 8.71 | |
| **Ramachandran plot** |  | |  | | |  | |
| Outliers (%) | 0 | | 0 | | | 0 | |
| Allowed (%) | 2.66 | | 1.47 | | | 1.13 | |
| Favored (%) | 97.34 | | 98.53 | | | 98.87 | |
|  | | | | | | | |
| Sample | **147-bp 5S rDNA nucleosome** | | | | | | |
| Structures | 147-bp 5S rDNA Nucleosome Closed | | | | 147-bp 5S rDNA Nucleosome Open I | | |
| Accession codes | PDB 9D3R; EMD-46545 | | | | PDB 9D3S; EMD-46546 | | |
| Type of support | Quantifoil R2/1, UT, 300 Mesh, Copper (Cat No. Q350CR1-2nm) | | | | | | |
| Microscope | Titan Krios G4 (Thermo Fisher Scientific) | | | | | | |
| Detector | Falcon 4 | | | | | | |
| Energy filter | Selectris X | | | | | | |
| Acceleration voltage (kV) | 300 | | | | | | |
| Pixel size (Å) | 0.935 | | | | | | |
| Total dose  (e^-^/Å^2^) | 50 | | | | | | |
| No. of micrographs recorded/used | 5571/5291 | | | | | | |
| Symmetry | C1 (symmetry not imposed) | | | | | | |
| No. of picked particles | 3,022,989 (100%) | | | | | | |
| No. of particles after 2D classifications | 2,239,232 (74%) | | | | | | |
| No. of particles after 3D classification with alignment | 208,470 (7%) | | | | | | |
| No. of particles in the final reconstruction after 3D classification without alignment | 16,369 (0.5%) | | | | 80,111 (3%) | | |
| Global resolution (Å) | 3.3 | | | | 3.1 | | |
|  | | | | | | | |
| **Refinement** |  | | | | | | |
| Model resolution (Å)  (FSC threshold 0.143) | 3.3 | | | | 3.1 | | |
| **Model composition** |  | | | |  | | |
| Chains | 10 | | | | 10 | | |
| Non-hydrogen atoms | 11858 | | | | 10939 | | |
| Protein residues | 750 | | | | 748 | | |
| Nucleotide | 290 | | | | 246 | | |
| **Mean B-factors (Å^2^)** |  | | | | | | |
| Protein | 58.63 | | | | 42.34 | | |
| Nucleotide | 142.43 | | | | 97.53 | | |
| **R.m.s. deviations** |  | | | | | | |
| Bond lengths (Å) | 0.008 | | | | 0.011 | | |
| Bond angles (°) | 0.889 | | | | 0.973 | | |
| **Validation** |  | | | | | | |
| MolProbity score | 1.82 | | | | 1.73 | | |
| Clash score | 12.72 | | | | 14.35 | | |
| **Ramachandran plot** |  | | | | | | |
| Outliers (%) | 0 | | | | 0 | | |
| Allowed (%) | 3.27 | | | | 2.32 | | |
| Favored (%) | 96.73 | | | | 97.68 | | |
|  | | | | | | | |
| Sample | **Glutaraldehyde (GA)-crosslinked 167-bp 5S rDNA nucleosome** | | **Formaldehyde (FA)-crosslinked 167-bp 5S rDNA nucleosome** | | | **Glutaraldehyde (GA)-crosslinked 147-bp 5S rDNA nucleosome** | |
| Cryo-EM map and model availability | PDB 9D3N; EMD-46540 | | EMD-46541 | | | PDB 9D3T; EMD-46547 | |
| Type of support | Quantifoil R2/1, UT, 300 Mesh, Copper (Cat No. Q350CR1-2nm) | | | | | | |
| Microscope | Titan Krios G4 (Thermo Fisher Scientific) | | | | | | |
| Detector | Falcon 4 | | | | | | |
| Energy filter | Selectris X | | | | | | |
| Acceleration voltage (kV) | 300 | | | | | | |
| Pixel size (Å) | 0.935 | | | | | | |
| Total dose  (e^-^/Å^2^) | 50 | | | | | | |
| No. of micrographs recorded/used | 5454/5152 | | 6443/6280 | | | 5622/5352 | |
| Symmetry | C1 (symmetry not imposed) | | | | | | |
| No. of picked particles | 1,939,825 (100%) | | 3,344,880 (100%) | | | 2,164,687 (100%) | |
| No. of particles after 2D classifications | 479,048 (25%) | | 1,493,665 (45%) | | | 1,076,644 (50%) | |
| No. of particles after 3D classification with alignment | 85,946 (4%) | | 308,514 (9%) | | | 184,144 (9%) | |
| No. of particles in the final reconstruction after 3D classification without alignment | 35,903 (2%) | | 224,003 (7%) | | | 53,805 (2%) | |
| Global resolution (Å) | 3.0 | | 2.6  (overestimated value due to preferential views) | | | 2.8 | |
|  | | | | | | | |
| **Refinement** |  | |  | | |  | |
| Model resolution (Å)  (FSC threshold 0.143) | 3.0 | | N/A | | | 2.8 | |
| **Model composition** |  | |  |  |  |  | |
| Chains | 10 | |  |  |  | 10 | |
| Non-hydrogen atoms | 9401 | |  |  |  | 9820 | |
| Protein residues | 697 | |  |  |  | 716 | |
| Nucleotide | 192 | |  |  |  | 206 | |
| **Mean B-factors (Å^2^)** |  | |  |  |  |  | |
| Protein | 46 | |  |  |  | 40.44 | |
| Nucleotide | 126.29 | |  |  |  | 97.83 | |
| **R.m.s. deviations** |  | |  |  |  |  | |
| Bond lengths (Å) | 0.003 | |  |  |  | 0.005 | |
| Bond angles (°) | 0.652 | |  |  |  | 0.729 | |
| **Validation** |  | |  |  |  |  | |
| MolProbity score | 1.58 | |  |  |  | 2.19 | |
| Clash score | 10.38 | |  |  |  | 10.36 | |
| **Ramachandran plot** |  | |  |  |  |  | |
| Outliers (%) | 0 | |  |  |  | 0 | |
| Allowed (%) | 220 | |  |  |  | 3.00 | |
| Favored (%) | 97.80 | |  |  |  | 97.00 | |

**Supplemental Table S1.** Cryo-EM data collection, refinement, and validation statistics.

**Supplemental Movie S1.** 3D variability analysis of the crosslinked Dsup-bound nucleosome after map refinement using an initial reference without one DNA end.

**Supplemental Movie S2.** 3D variability analysis of the crosslinked Dsup-bound nucleosome after map refinement using an initial reference without both DNA ends.

**Supplemental Movie S3.** 167-bp 5s rDNA nucleosome dynamics: downstream DNA opening.

**Supplemental Movie S4.** 167-bp 5s rDNA nucleosome dynamics: upstream DNA opening.
