# Supplementary material for: Structural basis of nucleosome recognition by the conserved Dsup and HMGN nucleosome-binding motif
Source: Genes Dev. 2025 Oct 1;39(19-20):1155–61. doi: 10.1101/gad.352720.125 (PMC12487698; doi:10.1101/gad.352720.125)
Supplement: Supplement 7 [file Supplemental_Table_S2.docx]

|  |  | **Sample** | | | | | |
| --- | --- | --- | --- | --- | --- | --- | --- |
|  |  | 167-bp 5S  rDNA Nucleosome | | 147-bp 5S rDNA Nucleosome | Glutaraldehyde (GA)-crosslinked 167-bp 5S rDNA Nucleosome | Formaldehyde (FA)-crosslinked 167-bp 5S rDNA Nucleosome | Glutaraldehyde (GA)-crosslinked 147-bp 5S rDNA Nucleosome |
| **5S rDNA Nucleosome Structural State Based on DNA Flexibility** | Closed  (Without Unwrapped Ends) | 5% | | 36% | 0% | 0% | 0% |
|  | One End Unwrapped | 95% | | 44%  (Open I-like) | 0% | 0% | 12% |
|  |  | 60%  (Open I-like) | 35%  (Open II-like) |  |  |  |  |
|  | Both Ends Unwrapped | 0% | | 0% | 100% | 100% | 73% |
|  | Heterogeneous Classes with Challenging Interpretation | 0% | | 20% | 0% | 0% | 15% |
|  | 3D Classification Without Alignment Input Particles | 100% | | 100% | 100% | 100% | 100% |

**Supplemental Table S2.** Percentage distribution of particles across 5S rDNA nucleosome structural states according to DNA flexibility. Particles selected after 2D classification and 3D classification with alignment were used in 3D classification without alignment and correspond to 100%.
